# p21-senescent cells drive pancreatic islet dysfunction through targetable paracrine signaling in type 2 diabetes

**DOI:** 10.1172/jci.insight.197310

**Published:** 2026-06-09

**Authors:** Kanako Iwasaki, Priscila Carapeto, Cristian Abarca, Francesko Hela, Stephanie Sanjines, Sebastian Pena, Sandra Le, Hui Pan, Maya Jackson, Christopher Cahill, Ayush Midha, Juliana Alcoforado Diniz, Dylan Baker, Sergii Domanskyi, Sara Espinoza, Alejandro Peña, Francisco G. Cigarroa, Jillian L. Woodworth, Jeffrey H. Chuang, Vesna D. Garovic, James L. Kirkland, Tamara Tchkonia, Nicolas Musi, George A. Kuchel, Paul Robson, Cristina Aguayo-Mazzucato

**Affiliations:** 1 Joslin Diabetes Center/Harvard Medical School, Boston, Massachusetts, USA.; 2 The Jackson Laboratory for Genomic Medicine, Farmington, Connecticut, USA.; 3 Center for Translational Geroscience, Department of Medicine, Cedars-Sinai Medical Center, Los Angeles, California, USA.; 4 UT Health San Antonio Transplant Center, San Antonio, Texas, USA.; 5 Department of Genetics and Genome Sciences, UConn Health, Farmington, Connecticut, USA.; 6 Division of Nephrology and Hypertension, Department of Medicine, Mayo Clinic, Rochester, Minnesota, USA.; 7 Center for Advanced Gerotherapeutics, Department of Medicine, Cedars-Sinai Medical Center, Los Angels, California, USA.; 8 UConn Center on Aging, UConn Health, Farmington, Connecticut, USA.

**Keywords:** Aging, Endocrinology, Metabolism, Beta cells, Cellular senescence, Diabetes

## Abstract

Cellular senescence is an irreversible stress response, which leads to loss of cellular function and remodeling of the cellular secretory profile. In humans, pancreatic β cells undergo cellular senescence during the progression to type 2 diabetes (T2D). However, the mechanism linking β cell senescence to islet dysfunction remains unknown, and thus the therapeutic potential of targeting senescent cells in T2D is not established. Herein, we identified a subpopulation of senescent β cells expressing p21, which emerged early in the progression of T2D in humans and mice. Spatial transcriptomics and proteomics analyses confirmed senescence and loss of cellular identity in this subpopulation in humans. Functional analysis revealed lack of glucose responsiveness, high basal insulin secretion, and transcription of senescence-associated secretory phenotype (SASP) factors. SASP factors from p21^+^ β cells induced secondary senescence in neighboring cells, characterized by dysfunction and loss of identity. JAK inhibitors counteracted the induction of secondary senescence and restored β cell function in islets from humans with T2D and in mice fed a high-fat diet. These findings reveal the critical role of p21^+^ β cells in T2D pathogenesis and the therapeutic potential of targeting this pathophysiological process.

## Abstract 2

A subset of senescent pancreatic β-cells impairs islet function by inducing secondary senescence in type 2 diabetes; JAK inhibitors may prevent this pathologic process.

## Introduction

As cells age or experience stress, they can enter a state of cellular senescence characterized by stable cell-cycle arrest, cellular dysfunction, resistance to apoptosis, and continued metabolic activity accompanied by secretion of senescence-associated secretory phenotype (SASP) factors. Beyond cell-autonomous effects, the SASP can induce secondary senescence in neighboring cells, thereby amplifying tissue dysfunction (1) and driving age-related diseases.

Insulin resistance and type 2 diabetes (T2D) are associated with increased senescence markers in mouse and human pancreatic β cells (2, 3). Senescent β cells are dysfunctional and secrete a distinct SASP enriched in inflammatory and extracellular matrix remodeling factors (3, 4). In mouse models, senolytic interventions improved insulin sensitivity, glucose homeostasis, and β cell identity and function while reducing SASP gene expression (3). However, whether β cell SASP factors propagate secondary senescence within pancreatic islets remains unknown. An alternative strategy to targeting senescent β cells is suppression of the SASP itself.

Notably, β cell senescence may not be uniformly detrimental. Some studies suggest senescence-associated programs can promote immune surveillance, cell survival, differentiation, and insulin secretion (5, 6). These apparently conflicting observations may reflect underlying heterogeneity among senescent β cells, consistent with the heterogeneous distribution of senescence markers within pancreatic islets (2). However, the identity, functional properties, and non-cell-autonomous effects of distinct senescent β cell populations remain poorly understood. Defining this heterogeneity will be essential for developing effective and safe therapeutic approaches for chronic diseases associated with β cell senescence, including T2D.

The cyclin-dependent kinase inhibitors p21 and p16 are major mediators of senescence across multiple cell types. These pathways, encoded by *Cdkn1a* and the *Cdkn2a* locus, respectively, are activated by diverse stressors including oncogene activation, telomere shortening, DNA damage, protein aggregation, and ROS (7).

Herein, senescent β cell heterogeneity is hypothesized to be driven by differential activation of the cell-cycle arrest pathways mediated by *Cdkn1a* (p21) and *Cdkn2a* (p16). We identified distinct senescent β cell subpopulations based on expression of these cell-cycle inhibitors. In C57BL/6 mice, *Cdkn1a*-expressing β cells represented the predominant senescent population and were characterized by canonical SASP activation, impaired β cell function, and loss of β cell identity. Spatial transcriptomic and proteomic analyses of human pancreas tissue revealed that *CDKN1A*/p21^+^ INS^+^ cells downregulated key β cell genes and transcription factors. Factors secreted by *Cdkn1a*^+^ β cells induced secondary senescence in neighboring islet cells, leading to impaired glucose-stimulated insulin secretion (GSIS) and loss of β cell identity. JAK1/2 inhibition prevented SASP-mediated secondary senescence and restored β cell function. In human islets from donors with and without T2D, JAK1/2 inhibitors (JAK1/2i) reduced p21 expression, suppressed SASP secretion, and improved glucose responsiveness. Together, these findings identify senescent β cell heterogeneity as a critical determinant of islet dysfunction and demonstrate that specific targeting of SASP signaling mitigates secondary senescence and restores β cell function in both mice and humans.

## Results

### p21^+^ senescent β cells in mice are dysfunctional and lose transcriptional identity.

**Figure 1:**
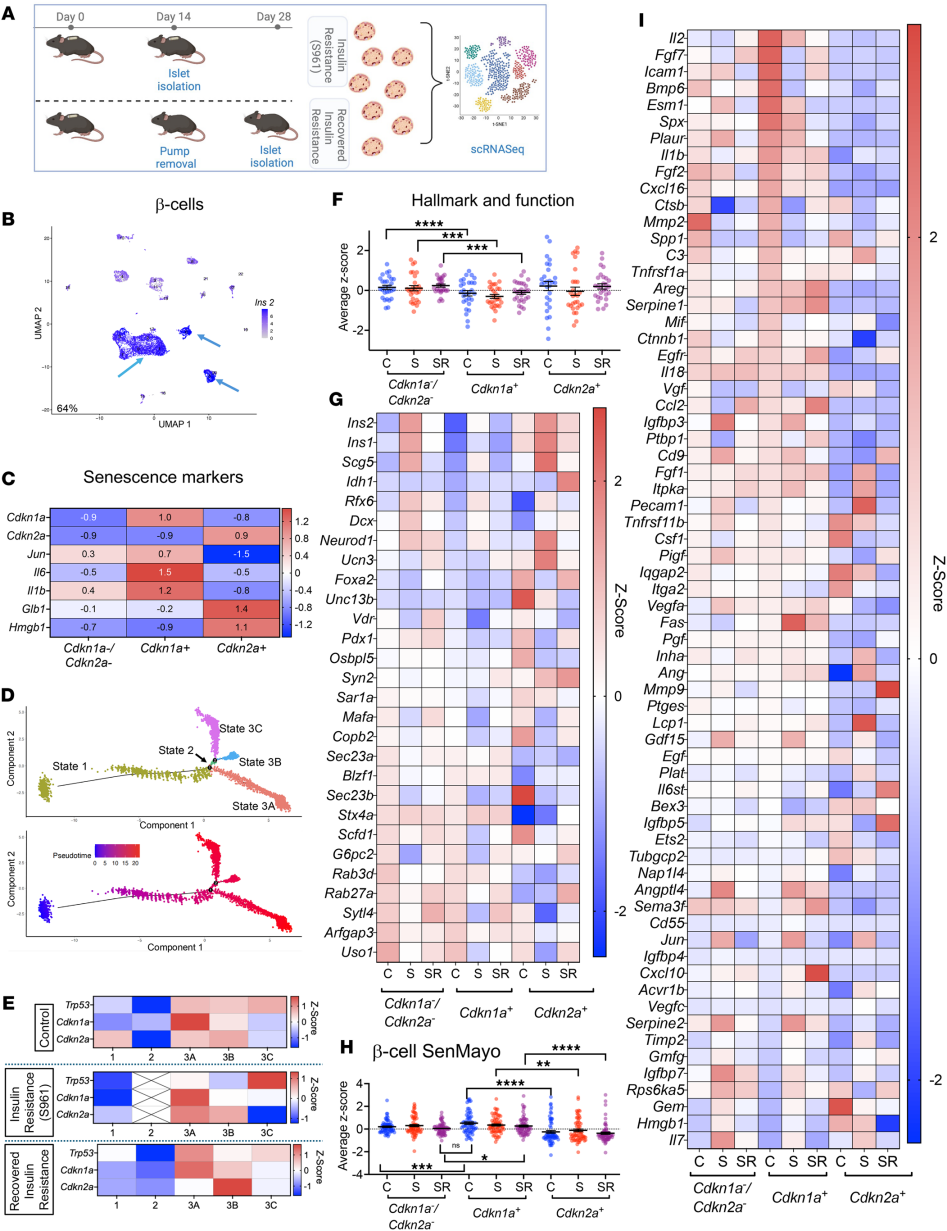
Subpopulations of senescent β cells with different functional and SASP transcriptional profiles. (**A**) Reanalysis of scRNA-Seq data (GSE149984) of islets isolated from C57Bl6/J male mice (7- to 8-month-old retired breeders) under 3 conditions: control (C), S961-induced insulin resistance for 2 weeks (S), and S961-induced insulin resistance followed by a 2-week recovery period (SR). (**B**) UMAP plot displaying the major islet-cell cluster of mouse β cells based on *Ins2* expression. (**C**) Heatmap of senescence marker genes with individual *z* scores. (**D**) Trajectory analysis scRNA-Seq from nonsenescent to senescent mouse β cells showing different stages along a pseudo-time-course and (**E**) specific senescent gene expression per metabolic condition. (**F**) Scatter plots of average *z* score expression levels of hallmark and functional genes in each subpopulation; mean ± SEM; expression levels analyzed by Wilcoxon’s matched-pairs signed-rank test. Data are results from 2,939 β cells in **C**, 2,896 β cells from S961R conditions, and 2,513 cells from SR; islets were isolated from 4 mice per condition as previously published (1). The midpoint of 0 represents average expression across all samples. (**G**) Heatmap of β cell hallmark and function genes. The graph shows the expression of the genes in 3 cell subpopulations of nonsenescent β cells (*Cdkn1a^–^/Cdkn2a^–^*) and *Cdkn1a^+^* and *Cdkn2a^+^* under the 3 metabolic conditions described. (**H**) Scatter plots of average *z* score expression levels of β cell SenMayo score. Mean ± SEM; expression levels analyzed by Wilcoxon’s matched-pairs signed-rank test. (**I**) β SenMayo: heatmap of selected genes related to senescence and SASP in the same 3 cell subpopulations under the different metabolic conditions. **P < 0.001, ***P < 0.0001, and ****P < 0.00001.

We used previously published scRNA-Seq data (4) to analyze gene expression in pancreatic islets from 6- to 9-month-old male mice after acute induction of insulin resistance with the insulin receptor antagonist S961, with and without a 2-week recovery period (Figure 1A), completely reversing hyperglycemia and hyperinsulinemia (Supplemental Figure 1A; supplemental material available online with this article; https://doi.org/10.1172/jci.insight.197310DS1). β Cells, identified by the expression of *Ins2*, represented 64% of the total islet population in the control group (Figure 1B). Other islet cell types were represented in the following proportions: α 11%, δ 9%, pancreatic polypeptide 2%, ductal 2%, and endothelial 5% (Supplemental Figure 2). Density estimates for the expression of *p21* (encoded by *Cdkn1a*) or *p16* (encoded by *Cdkn2a*) using a Gaussian finite mixture model identified at least 2 subpopulations of senescent β cells: *Cdkn1a*^+^ and *Cdkn2a*^+^, as well as a third nonsenescent subpopulation of double-negative *Cdkn1a*^–^/*Cdkn2a*^–^ cells. Nonsenescent (*Cdkn1a^–^/Cdkn2a^–^*) β cells represented 75% of the total population of control islets. The remaining senescent cell subpopulations included 29% *Cdkn1a*^+^ cells and 0.3% *Cdkn2a*^+^ cells (Supplemental Figure 3). These proportions align with reported values of 20%–40% of *CDKN1A*^+^ β cells from adults over 60 years old (8). The frequency of the senescent subpopulation in other islet cell types is shown in Supplemental Table 1.

To determine whether the upregulation of *Cdkn1a* and *Cdkn2a* represented true senescence identities, coexpression with other senescence markers in the various subpopulations was evaluated (Figure 1C). The *Cdkn1a*^+^ subpopulation coexpressed cell proliferation regulator *Jun* and the SASP factors *Il6* and *Il1b,* while the *Cdkn2a*^+^ subpopulation coexpressed *Glb1,* which encodes senescence marker β-gal, and senescence marker *Hmgb1*. These results support the notion that these 2 subpopulations are in a state of senescence and are distinct from each other.

With insulin resistance, both the *Cdkn1a*^+^ and *Cdkn2a*^+^ senescent β cell subpopulations increased (Supplemental Figure 3). The *Cdkn1a*^+^ subpopulation decreased during recovery, while the number of *Cdkn2a*^+^ cells continued to increase (Supplemental Figure 3), indicating distinct dynamics and discrete populations. Principal component analysis (PCA) of the *z* scores revealed significant clustering by treatment (Supplemental Figure 4); therefore, results are shown for all 3 treatments in each senescent subpopulation. Trajectory analysis of scRNA-Seq from nonsenescent to senescent mouse β cells revealed 5 stages based on transcriptional similarity, 3 of which appear to be alternate senescence fates, which we have named stage 3, A–C, with states 1 and 2 being nonsenescent (Figure 1D). Characteristically, in β cells from all metabolic conditions, stage 3A had the highest *Cdkn1a* levels, stage 3B was characterized by *Cdkn2a* expression, and stage 3C had high *Trp53* levels, suggesting a stressed cell fate (Figure 1E). *Cdkn1a*^+^ β cells in all metabolic conditions had downregulated expression of hallmark β cell genes, including *Ins1, Ins2, and Pdx1*, as well as functional genes under all metabolic conditions (Figure 1, F and G, and Supplemental Figure 5A). Interestingly, this set of functional genes was not downregulated in senescent *Cdkn2a*^+^ β cells (Figure 1, F and G, and Supplemental Figure 5A), revealing potential functional differences between subpopulations of senescent β cells and reconciling previous reports of increased functionality after *p16* (*Cdkn2a*) overexpression in β cells (5). Distinct *Cdkn1a*^+^ and *Cdkn2a*^+^ subpopulations have also been identified in other tissues, including adipose tissue, liver, and heart (9).

Expression of SASP factors in different senescent subpopulations included genes published in the SenMayo panel (10). To minimize artifacts introduced by specific gene dropout, only genes with quantifiable reads in all 3 subpopulations were included in the analysis and reported as β-SenMayo (Figure 1, H and I, and Supplemental Figure 5B). Compared with nonsenescent and *Cdkn2a*^+^ cells*,* the *Cdkn1a*^+^ subpopulation had a significantly higher β-SenMayo score with increased transcripts of *Il1b*, *Plaur*, *Gdf15*, and *Fgf1* (Figure 1I and Supplemental Figure 5B), further corroborating its senescent phenotype.

**Figure 2:**
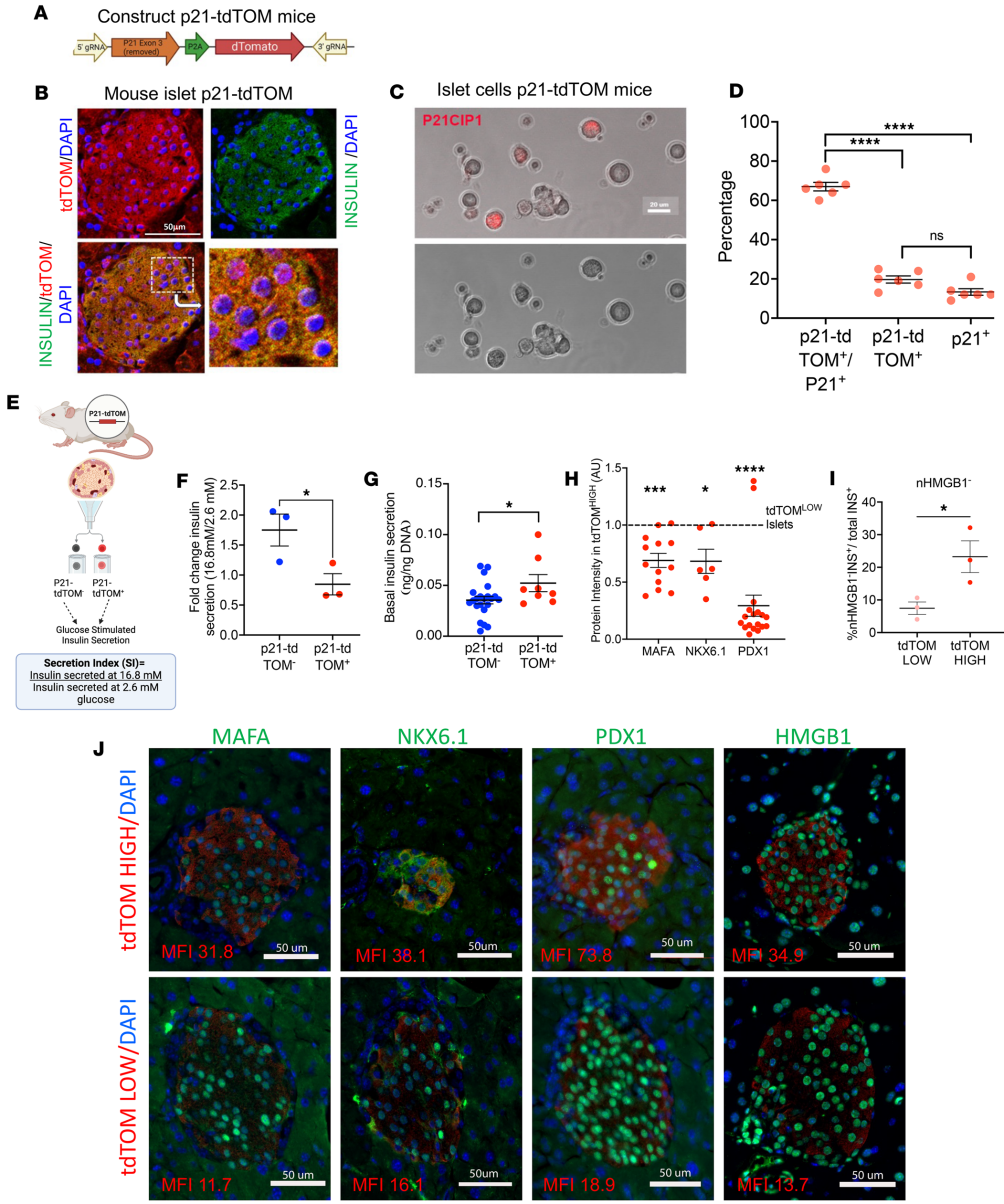
The p21^+^-β cell subpopulation was dysfunctional and lost its identity. (**A**) P2A-dTomato transgene used in the generation of *p21-*tdTomato mice, inserted at the end of the coding region using CRISPR. (**B**) Panels of different confocal microscopy channels reveal coexpression of insulin and *p21* in islet cells; inset shows its nuclear localization. Representative image shown from *n* = 7 mice. Scale bar: 50 μm. (**C**) Live fluorescence with bright-field microscopy of dispersed islets isolated from 3-month-old *p21-*tdTomato (scale bar: 20 μm) and bright-field channel only showing live islet cells. (**D**) The proportion of tdTomato and p21 in islets analyzed by FACS from 6 tdTomato mice (male and female, 40–65 weeks). Each dot represents 1 mouse. ****P < 0.0001, 1-way ANOVA. (**E**) Islet isolation, dissociation, and sorting based on tdTomato signal to purify P21-tdTOM^+^ and P21-tdTOM^–^ for functional analysis. (**F**) Secretion index = ratio of insulin secreted at 16.8 mM glucose compared with 2.8 mM glucose; *n* = 3 biological replicates; *P < 0.05 by 2-way paired *t* test. (**G**) Basal insulin secretion in P21-tdTOM^+^ and P21-tdTOM^–^ cells separated by flow cytometry; *n* = 2–5 technical replicates from 3 biological replicates. Mean ± SEM; islets isolated from 4 male mice (53–70 weeks) and 3 female mice (51–72 weeks). *P < 0.05 by 2-way unpaired *t* test. (**H**) Loss of β cell identity at the protein level. Semiquantification of protein intensity in confocal immunofluorescent images from tdTOM^hi^ islets with respect to tdTOM^lo^. Each point represents an individual islet from 3 P21-tdTOM^+^ female mice (17–22 weeks). (**I**) HMGB1 nuclear exclusion frequency in INSULIN^+^ cells in islets from tdTOM^hi^ and tdTOM^lo^. Each point represents an individual islet from 3 P21-tdTOM^+^ female mice (17–22 weeks). (**J**) Representative images of **H** and **I** comparing protein expression of β cell hallmark genes in islets from tdTOM^hi^ and tdTOM^lo^. tdTomato in red; β cell–related genes and HMGB1 in green. MFI for red channel intensity. Scale bar: 50 μm.

Validation of the senescent *p21* cell subpopulation at the protein expression level included the development of a *p21-*tdTomato (*p21-*tdTom) reporter mouse model. *p21-*tdTom mice were generated by CRISPR/Cas9-mediated knockin of a P2A-tdTomato cassette into the 3′ end of the endogenous *p21* gene in a bicistronic fashion, leading to the production of 2 proteins, P21 and tdTomato, from the same transcript (Figure 2A). Metabolic evaluation of the p21-tdTom reporter showed no difference between sexes and with respect to WT mice in glucose clearance (Supplemental Figure 6, A and B). Doxorubicin treatment increased *Cdkn1a* expression and decreased *Pdx1* expression in both islets from reporter and non-reporter mice (Supplemental Figure 6, C and F), showing that gene expression was not affected by the transgene construct (Supplemental Figure 6, H and I). Insulin content was also not affected by the reporter (Supplemental Figure 6J). Pancreatic islets isolated from these mice had detectable tdTom-expressing β cells, as shown by IHC (Figure 2B) and live fluorescence microscopy (Figure 2C), confirming effective protein translation. There was a 67% overlap between p21 protein by immunostaining and tdTom red signal by flow cytometry (Figure 2D and Supplemental Figure 7).

To test the age dependency of the p21 subpopulation, islets from young (3 months old) and aged (18 months old) *p21-*tdTom mice were dispersed into single cells. p16 was detected by immunostaining, and p21 was detected by endogenous Tomato fluorescence. All 3 subpopulations detected in the scRNA-Seq data were also detected at the protein level in islets from 3-month-old mice (Supplemental Figure 8) in the following proportions: 18% p21^+^ and 4% p16^+^, supporting the scRNA-Seq results. The p21^+^ subpopulation significantly increased with age to 28%.

The function of the *p21^+^* subpopulation was evaluated by isolating and dispersing islets from aged adult male and female *p21-*tdTom mice (51–72 weeks of age). Flow cytometry was used to separate cells into *p21*-tdTOM*^–^* and *p21*-tdTOM^+^ subpopulations, and the response to glucose was evaluated with GSIS (Figure 2E). Senescent *p21*-tdTOM^+^ cells had impaired glucose responsiveness, as shown by a decreased insulin secretion index (insulin secretion at 16.8 mM glucose/insulin secretion at 2.6 mM glucose) (Figure 2F) due to increased basal insulin secretion (Figure 2G), a feature typical of dysfunctional β cells (2, 3, 11).

To assess whether the decreased expression of genes related to β cell identity and function observed in the *Cdkn1a*^+^ subpopulation was reflected at the protein level, pancreatic sections from *p21-*tdTom mice were stained for the following antigens and their intensity quantified: MAFA, PDX1, and NKX6.1. The results showed a significant decrease in the protein levels of these β cell transcription factors in islets with high levels of tdTOM compared with islets with low levels of tdTOM, as assessed by semiquantitative immunofluorescence (Figure 2, H and J), confirming loss of cellular identity. Furthermore, HMGB1, another marker of cellular senescence, showed predominant nuclear exclusion in islets with high tdTOM levels (Figure 2, I and J), supporting its senescent identity.

In summary, these analyses identified a *Cdkn1a*^+^ (encoding *p21*) β cell subpopulation that exhibited low glucose responsiveness, loss of β cell identity, and increased expression of well-known senescence and SASP genes.

### Non-cell-autonomous effects of the β cell SASP induce secondary senescence.

To assess whether SASP factors from senescent β cells had nonautonomous effects on nonsenescent β cells and contribute to senescence, a series of experiments were conducted using complete conditioned media (CM) containing either the full SASP from senescent cells, a selection of 4 SASP factors, or individual SASP factors.

**Figure 3:**
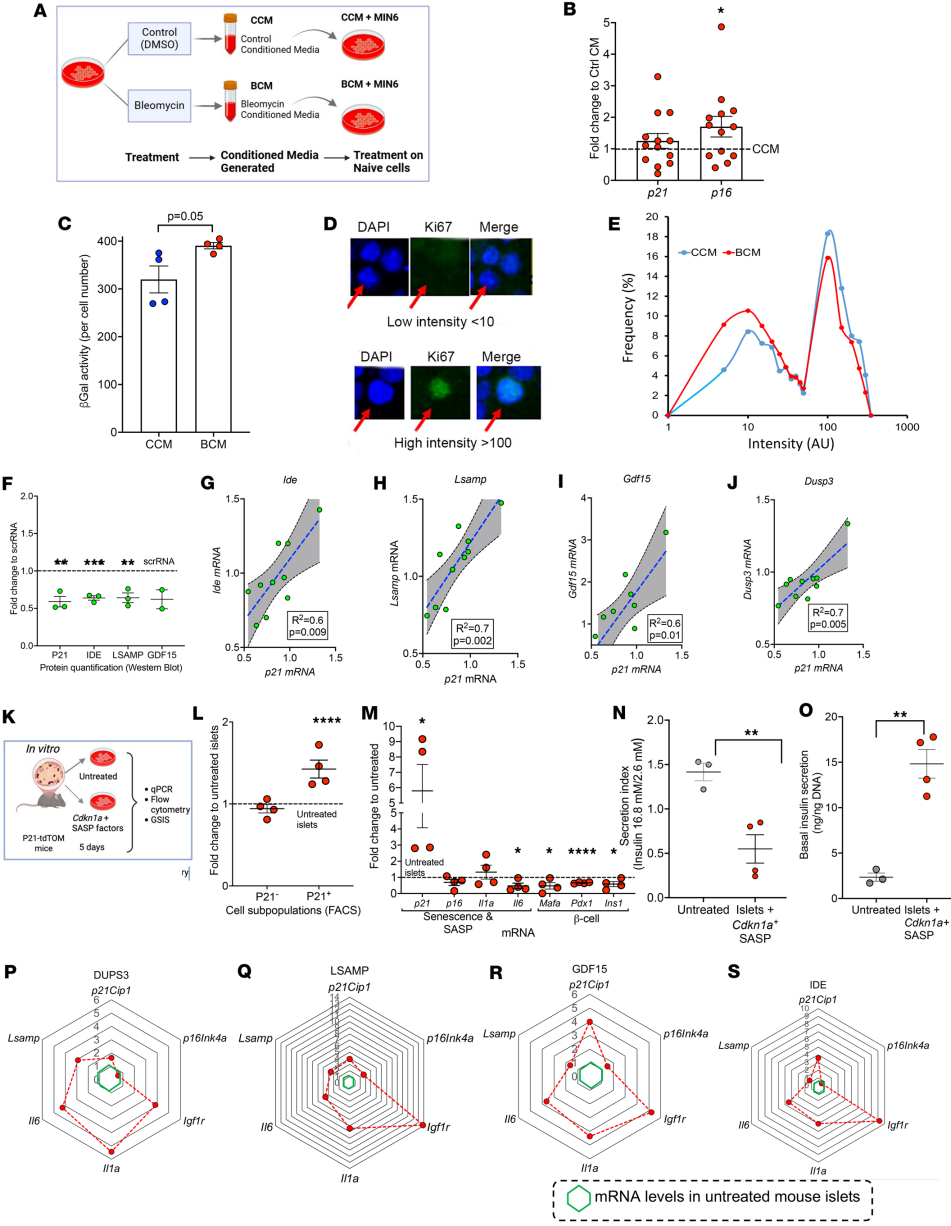
Secondary senescence: β cell SASP has non-cell-autonomous effects on neighboring cells. (**A**) Workflow to test the effects of SASP on naive MIN6 cells. (**B**) Effects of BCM on *p21* and *p16* mRNA expression of naive MIN6 cells (*n* = 13–14 technical replicates from 3 biological replicates) *P < 0.05, analyzed by nonparametric Wilcoxon’s test. (**C**) β-Gal^+^ activity normalized per cell number (*n* = 4 biological replicates). (**D**) Cumulative frequency graph of Ki67 staining of MIN6 cells reflecting proliferating subpopulations (cells counted: *n* = 2,911 for control CM, *n* = 3,000 for BCM (4 technical replicates in each of 2 biological replicates). (**E**) Protein quantification by Western blot of selected *Cdkn1a*^+^ SASP factors after treatment with p21 siRNA at 50 nM in MIN6 cells (*n* = 3 biological replicates). (**F**–**I**) Expression levels of *Cdkn1a*-SASP factors from individual samples and their correlations with *Cdkn1a* expression. Lines of best fit are shown, along with dotted lines indicating 95% CI. *P* values were calculated using the null hypothesis that the slope of the best fit line equals 0. (**J**) Treatment of islets from p21-tdTom mice with *Cdkn1a* SASP factors (LSAMP^+^DUSP3^+^GDF15^+^IDE) during 5 days (female and male, 12–20 weeks old). (**K**) Cellular subpopulations as determined by flow cytometry of dispersed islets after treatment. (**L**) Effects of *Cdkn1a^+^* SASP factors on transcription of selected senescence and key β cell genes. (**M**) Secretion index of islets treated with *Cdkn1a^+^* SASP factors. (**N**) Basal insulin secretion (*n* = 4 biological replicates). Mean ± SEM; expression levels analyzed by 2-way *t* tests. (**O**–**R**) Radar plots of qPCR results for the mean expression of senescence and SASP genes. MIN6 cells treated with an individual SASP. Mean ± SEM; *n* = 2 biological replicates with 2–3 technical replicates each. Concentrations of SASP proteins used in culture media are shown in Table 1. Data shown as mean ± SEM; expression levels analyzed by 2-way *t* tests; *P < 0.01, **P < 0.001, ***P < 0.0001, and ****P < 0.00001.

For complete CM experiments, a mouse insulinoma cell line, MIN6, was treated with CM from senescent and nonsenescent cells. CM from senescent cells was generated by treating MIN6 cells with the DNA-damaging agent, bleomycin, which induces senescence and SASP secretion in vitro (4). CM from vehicle-treated cells (control CM) or bleomycin-treated cells (BCM) was collected and used to treat naive MIN6 cells for 24 hours. Five days after exposure to control CM or BCM, cells were collected, and expression of senescence markers and proliferation were analyzed (Figure 3A). Exposure to BCM resulted in increased *p16* expression (Figure 3B) and a marginal increase in β-gal activity (Figure 3C), both of which are senescence markers (12).

Proliferative arrest, an additional indicator of senescence, was analyzed by measuring Ki67 staining intensity in individual nuclei using ImageJ (NIH). A frequency distribution graph of Ki67 intensity revealed 2 main subpopulations: nonproliferative cells (*Ki67* intensity < 10 AU, as measured with image analysis software) and proliferative cells (Ki67 intensity > 100 AU) (Figure 3, D and E). MIN6 cells treated with BCM had a greater percentage of nonproliferating cells and a lower percentage of proliferating cells, suggesting induction of senescence. These findings support a non-cell-autonomous effect of the SASP, capable of inducing secondary senescence.

To analyze non-cell-autonomous effects of individual SASP in β cells, 4 factors were selected based on the following criteria: (a) previously published as a senolytic target and/or SASP factor; (b) identified as a SASP factor secreted by β cells in our proteomic analysis (4); (c) common factor between mouse and human SASP and/or reported increase in β cells from donors with T2D (4); and (d) measured concentration in plasma by immunoassay as reported in the Human Protein Atlas (Table 1 and Supplemental Table 2). The following SASP factors met these criteria and were preferentially transcribed by *Cdkn1a*^+^ cells: LSAMP, IDE, DUSP3, and GDF15.

To test the dependency of these SASP factors on *p21*, a conditional siRNA knockdown was developed. At the protein level, conditional downregulation of *p21* led to significant decreases in IDE and LSAMP (Figure 3F). At the transcriptional level, a significant positive correlation was observed between *Lsamp*, *Dusp3*, *Gdf15*, and *Ide,* and the mRNA levels of *p21* (Figure 3, G–J). These patterns show that the chosen SASP factors directly correlated with the CDK inhibitor *p21*, thereby supporting subpopulation specificity.

To test the non-cell-autonomous effects of *p21*-SASP factors, islets were isolated from male and female *p21*-tdTOM mice and incubated with a combination of *Cdkn1a*^+^ SASP factors (LSAMP, DUSP3, GDF15, and IDE) for 5 days before assessing senescence and function (Figure 3K). Protein concentration levels were determined by reported circulating levels in humans in the Human Protein Atlas; however, the paracrine concentrations are likely higher than those reported in plasma but impossible to measure with currently available technology. SASP from *Cdkn1a*^+^ cells significantly increased the p21 subpopulation as measured by flow cytometry (Figure 3L and Supplemental Figure 7) and upregulated *p21* mRNA (Figure 3M), while downregulating the key β cell genes *Mafa, Pdx1*, and *Ins1*. At the functional level, *p21*-SASP factors impaired β cell function, as reflected by a lack of glucose responsiveness (Figure 3N), characterized by increased basal insulin secretion (Figure 3O).

To test whether individual *p21*-SASP factors were able to induce secondary senescence, MIN6 cells were treated with individual proteins for 4 days. All 4 proteins upregulated expression of 6 senescence and SASP genes (*p21*, *p16*, *Igf1r*, *Il1a*, *Il6*, and *Lsamp*) (Figure 3, P–S); LSAMP was the individual factor that induced the greatest increases in expression of senescence genes (Figure 3Q).

These results indicate that SASP factors induced secondary senescence in islet cells in a pathway-specific way: SASP released from p21^+^ cells increased the p21 subpopulation. SASP factors induced senescence both in combination and individually, implying that a small percentage of senescent p21^+^ β cells can induce secondary senescence and loss of β cell identity. Based on these findings, the β cell SASP can be considered an additional therapeutic target for restoring β cell identity and functionality.

### siRNA-mediated p21 knockdown improves insulin secretion.

**Figure 4:**
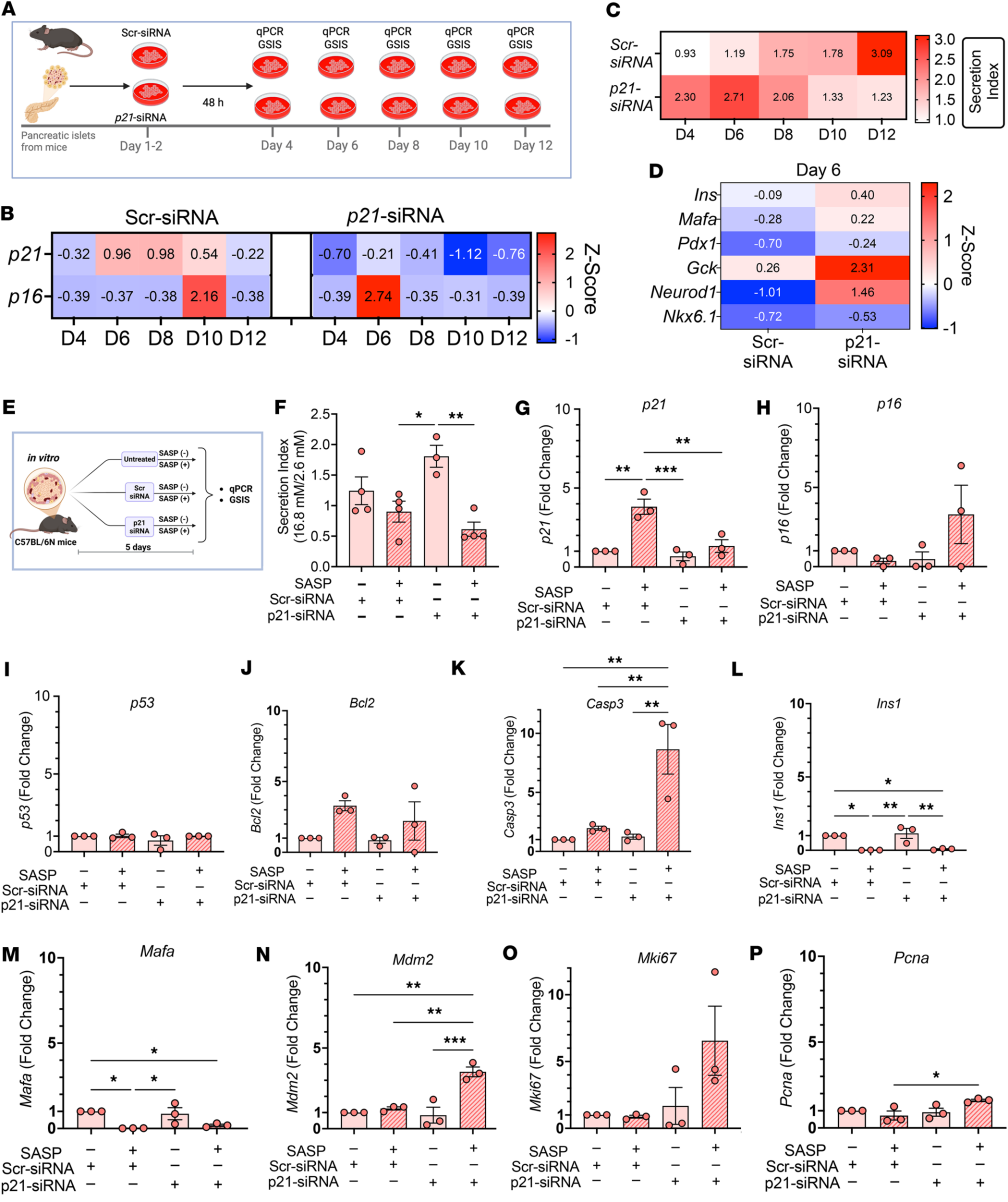
*Cdkn1a^+^* SASP factors induce secondary cellular senescence in mouse pancreatic islets in vitro. (**A**) Experimental design of the conditional *p21* knockdown time-course on mouse islets to assess effects on transcription and function. (**B**) Time-course expression levels of *p21* and *p16* mRNA under control (Scr-siRNA) and conditional p21 knockdown (p21-SiRNA, 50nM). (**C**) Time-course of β cell function as reflected by secretion index at different days after conditional knockdown of *p21*. (**D**) β cell hallmark and function genes after *p21* conditional knockdown (p21-siRNA) at day 6; 100 islets per condition from retired male breeders, *n* = 7 biological replicates; data shown as heatmap of *z* score for **B** and **D**, and of secretion index for **C**. (**E**) Experimental design for the evaluation of *p21*-SASP factors after *p21* conditional knockdown (p21-siRNA) in mouse islets. (**F**) Secretion index from static GSIS from *p21*-siRNA or *Scr*-siRNA with/without SASP factors (LSAMP^+^DUSP3^+^GDF15^+^IDE) during 5 days. Islets from 33 male C57Bl/N mice were used (6–9 months). (**G**–**P**) Transcriptomic gene levels from same experiment design using islets from 24 female and male C57Bl/N mice (17 weeks to 9 months). Mean ± SEM, 1-way ANOVA, *P < 0.05, **P < 0.01, ***P < 0.001.

To evaluate the temporal patterns of *p21* and *p16* expression in β cells, mouse pancreatic islets were isolated, and a time-course expression was delineated during 12 days in culture (Figure 4A). In control (Scr-siRNA) conditions (Figure 4B), *p21* increased from days 6–10; *p16* peaked later, at day 10. When *p21*-siRNA was used to keep levels down, the *p16* peak occurred earlier, at day 6 (Figure 4B). This timeframe is consistent with loss of identity and function in *p21*^+^ β cells, since the conditional knockdown improved β cell function (Figure 4C) and increased expression of key β cell genes at day 6 (Figure 4D). To examine the role of *p21* and *p16* in the induction of secondary senescence, these genes were knocked down with siRNA in islet cells exposed to *p21*-SASP factors (LSAMP, DUSP3, GDF15, IDE) (Figure 4E and Supplemental Figure 9A). From a functional perspective, *p21*-siRNA improved β cell function under basal conditions but did not rescue the functional loss induced by SASP (Figure 4F), *Ins1* nor *Mafa* transcription (Figure 4, L and M), suggesting additional *p21*-indedpendent pathways leading to functional and identity loss in secondary senescence. From a transcriptional perspective, knocking down *p21* significantly decreased *p21*-SASP–induced expression (Figure 4G) with no significant changes in *p16* expression (Figure 4H). Instead, *Mdm2* (p53 negative regulator) (Figure 4N) and *Casp3* (apoptosis) (Figure 4K) were upregulated. Proliferation genes *Mki67* and *Pcna* showed the same trend (Figure 4, O and P). No transcriptional changes of *p53* (Figure 4I) and *Bcl2* (Figure 4J) were detected. Overall, these changes suggest that in the absence of *p21*, SASP factors induce alternate cell fates such as apoptosis and/or proliferation. These possibilities should be explored further.

When *p16* was knocked down in islets exposed to *p21*-SASP factors (Supplemental Figure 9, A and B), there was an increase in *p21* expression (Supplemental Figure 9C), with no significant changes in other senescence markers: *p53, Bcl2,* and *Mdm2* (Supplemental Figure 9, D–F). These results suggest that in the absence of *p16*, senescence might be induced through the *p21* pathway.

### JAKi restored β cell function, transcriptional identity, and cell subpopulations in mouse islets.

**Figure 5:**
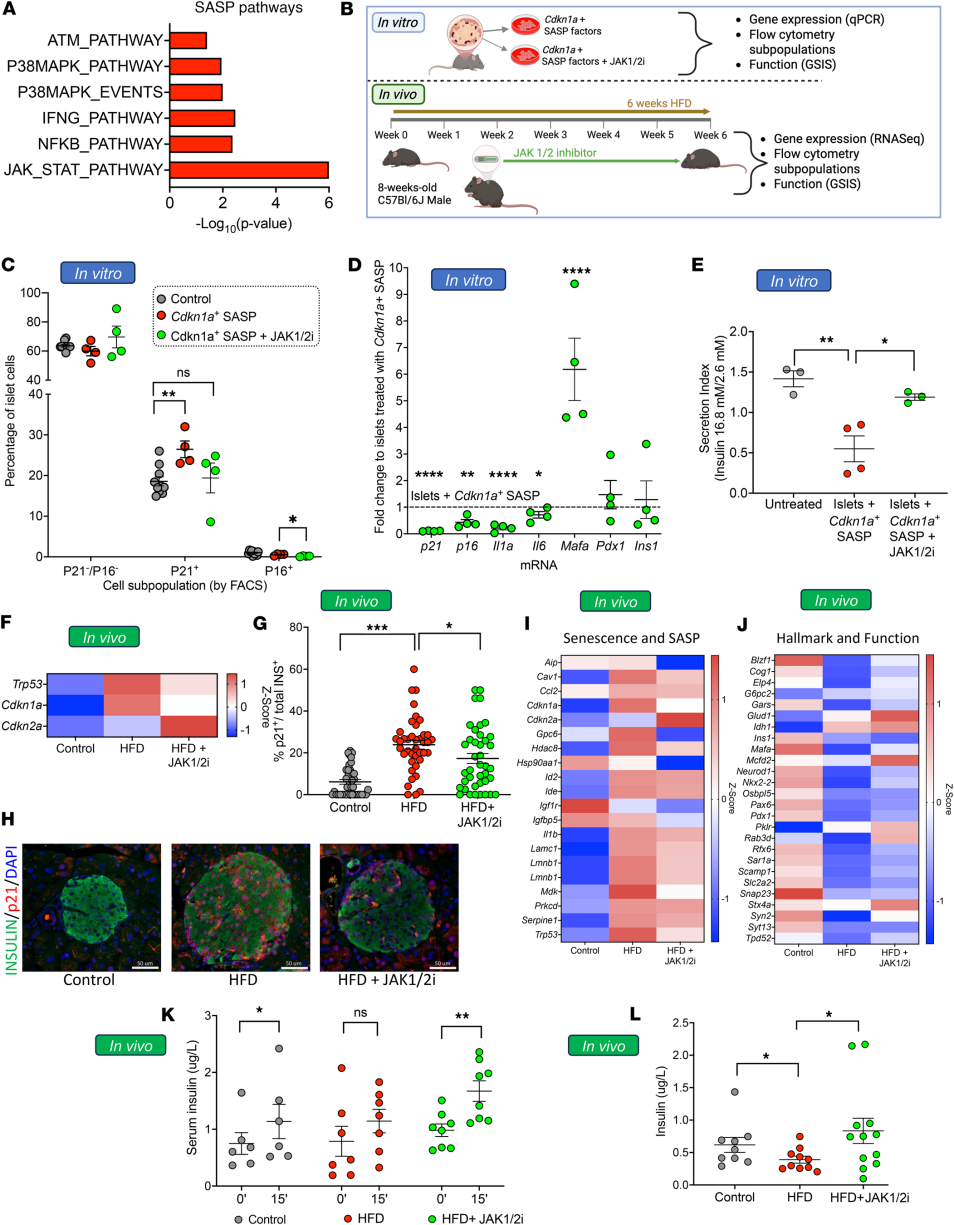
Secondary senescence induced by *Cdkn1a^+^* SASP factors is counteracted by JAK1/2i in mouse islets in vitro and in vivo. (**A**) Pathway analysis of SASP-regulating pathways in RNA-Seq data from mouse senescent β cells (6) reveals upregulation of the JAK/STAT pathway. (**B**) Experimental design in vitro: Treatment of islets from p21-tdTom mice with *Cdkn1a* SASP factors (LSAMP^+^DUSP3^+^GDF15^+^IDE) in combination with JAK1/2i. In vivo: male C57Bl/6N mice were fed a high-fat diet (HFD) for 6 weeks and treated for 4 weeks with JAK1/2i. *n* = 9 control, *n* = 11 HFD group, *n* = 13 HFD + JAK1/2i. (**C**) Flow cytometry analysis of dispersed islets treated with *Cdkn1a* SASP factors with or without JAK1/2i. (**D**) Transcriptional analysis of islets treated with *Cdkn1a* SASP factors with or without JAK1/2i. (**E**) Functional analysis of islets treated with *Cdkn1a* SASP factors with or without JAK1/2i. *n* = 4 independent experiments. (**C**–**E**) Mean ± SEM; expression levels analyzed by 2-way *t* tests with respect to control; *P < 0.01, **P < 0.001, and ****P < 0.00001. (**F**) Heatmap showing expression of key senescent genes in islets isolated from different treated groups. (**G**) Percentage of p21 (red) positive cells in INSULIN (green) positive cells determined by semiquantitative IHC in 3 groups: control, HFD, and HFD+JAK1/2i. Four 16–24-week-old female p21-tdTom mice were used in each group, and 933–1,245 islets were counted per group. *P < 0.05, ***P < 0.0001 by ordinary 1-way ANOVA followed by Tukey’s multiple comparisons test. (**H**) Representative images of islets immunostained for p21 (red) positive cells in INSULIN (green) in different treatment groups. Scale bar: 50 mm. (**I**) *z* score expression of senescence and SASP genes. (**J**) *z* score expression of β cell hallmark and functional genes. (**K**) GSIS by sampling plasma at minutes 0 and 15 during the GTT (i.p.). Mean ± SEM; *P < 0.01, **P < 0.001 by 2-way paired *t* test. (**L**) Fed plasma insulin in mice. Mean ± SEM; *P < 0.01 by 2-way unpaired *t* test.

SASP secretion and action are known to be regulated by several pathways (13), including the bromodomain and extra-terminal domain (BET) pathway (14), whose regulation was queried in mouse senescent β cells (3). A significant and prominent upregulation of the JAK/STAT pathway (Figure 5A and Supplemental Figure 10) was identified compared with nonsenescent β cells, and JAKi were tested in vitro and in vivo (Figure 5B) to assess their efficacy in maintaining β cell function and identity while decreasing SASP and secondary senescence.

In vitro, JAK1/2i attenuated the effects of *p21*-SASP factors by decreasing the p21 and p16 subpopulations of senescent cells measured by flow cytometry (Figure 5C and Supplemental Figure 7). At the transcriptional level, JAK1/2i decreased all tested senescence and SASP genes in islets treated with *p21*-SASP factors (Figure 5D), while upregulating *Mafa*, a key transcription factor for β cell function. These positive transcriptional and subpopulation changes were reflected at the functional level. Concurrent treatment of mouse islets with *p21*-SASP factors and JAK1/2i restored the secretion index, indicating improved β cell functionality (Figure 5E).

The effects of JAKi were also evaluated in vivo with a model of insulin resistance in which mice were fed a high-fat diet (HFD) that increases senescence in β cells (3) and other metabolically relevant tissues (15). Young (8-week-old) *C57Bl/6N* male mice were fed an HFD during 6 weeks. They received an osmotic pump containing vehicle or a JAK1/2i for the last 4 weeks (Figure 5B). Downregulation of the JAK pathway was confirmed at a transcriptional level (*P* < 0.02), with no differences in the SMAD/activin pathway, which can be modulated by JAKi. Bulk RNA-Seq of islets isolated from animals in the different treatment groups showed *Cdkn1a* induction by the HFD (Figure 5F). Also, the HFD increased p21 protein levels, which were suppressed by treatment with JAK1/2i (Figure 5, G and H). At the transcriptomic levels, increased expression of senescence and SASP genes (Figure 5I) and decreased expression of β cell hallmark identity and functional genes (Figure 5J) were observed with the HFD, which were restored by JAK1/2i. Functionally, JAK1/2i restored glucose responsiveness lost after the HFD in vivo, as evaluated during an i.p. glucose tolerance test (GTT) (Figure 5K), and increased circulating insulin levels (Figure 5L).

The peripheral effects of JAK1/2i were evaluated using qPCR in the following metabolically relevant tissues: liver, visceral adipose tissue, and skeletal muscle (Supplemental Figure 11). JAK 1/2i did not have a significant effect on the expression of senescence or SASP genes at the doses used (12.5 mg/kg/day), suggesting a preferential effect on β cells.

These results showed that JAKi decreased senescence genes and subpopulations while restoring β cell function and transcriptional identity in mouse β cells, both in vitro and in vivo.

### Human 21^+^ β cells are dysfunctional and lose transcriptional identity.

**Figure 6:**
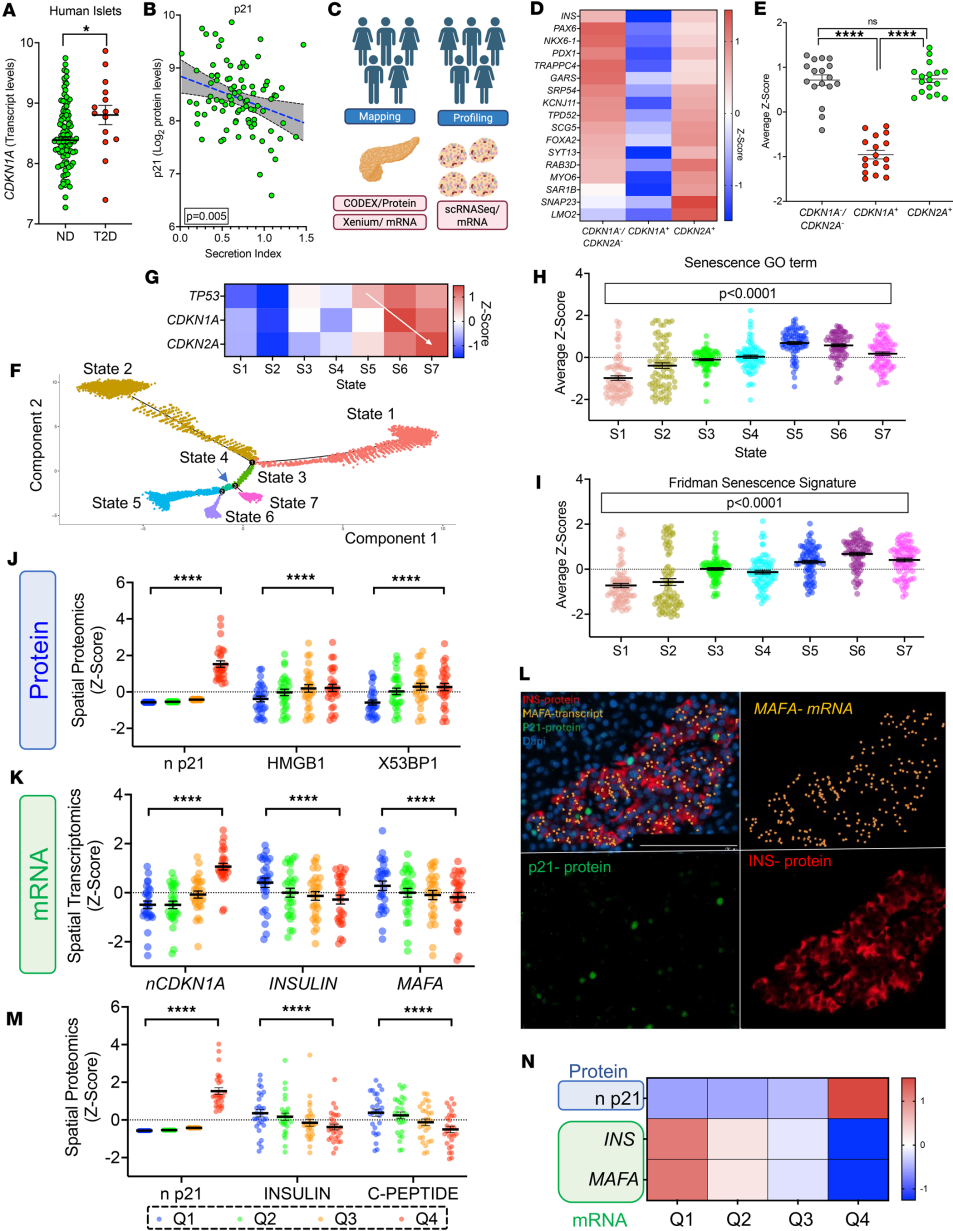
Loss of function and identity in human p21^+^ β cells. (**A**) *CDKN1A* mRNA levels in islets from human donors without diabetes (ND) and with T2D. (**B**) Linear correlation between p21 protein levels in human islets and their function as expressed by the secretion index. (**C**) KAPP-Sen study design aimed at mapping and profiling senescent cells in both whole and dispersed human pancreas. (**D** and **E**) Heatmap and dot plot of human β cell scRNA-Seq data for selected β cell functional genes across 3 cell subpopulations: nonsenescent β cells (*CDKN1A^–^/CDKN2A^–^*), *CDKN1A^+,^* and *CDKN2A^+^*. (**F**) Pseudo-time trajectory analysis of scRNA-Seq from nonsenescent to senescent human β cells. (**G**) Heatmap showing expression levels of key senescence genes at various stages during the senescence trajectory. (**H**) scRNA-Seq expression levels of genes in the senescence Gene Ontology (GO) category and (**I**) Fridman gene senescent scores. Data shows individual cells in each trajectory state, mean ± SEM; expression levels analyzed via 2-way ANOVA. Quartiles of nuclear P21 protein expression derived from spatial proteomic (CODEX) data, only from β cells. (**J**) Coexpression of senescence proteins within cells exhibiting quartile distribution of nuclear P21. (**K**) Coexpression of β cell transcripts within cells exhibiting quartile distribution of nuclear P21. (**L**) Representative image of integrated CODEX (INSULIN [red] and p21 [green] protein) with Xenium (*MAFA* [orange] transcript). (**M**) Quartiles of nuclear *CDKN1A* mRNA expression from Xenium and coexpression with hallmark β cell genes. (**N**) Heatmap showing overlapping coexpression of nuclear P21 with β cell hallmark genes.

Analysis of the relevance of the *p21*^+^ β cell population to human islets and disease included data from the Human Islets Database (16). *CDKN1A* transcript levels were significantly increased in islets from donors with T2D compared with islets from donors without diabetes (Figure 6A). Additionally, there was a negative correlation between P21 protein levels and the insulin secretion index (Figure 6B), suggesting that human p21^+^ β cells are also dysfunctional.

To further profile and map *p21*^+^ β cells in human islets, whole pancreas and human islets were obtained as part of the SenNet KAPP-Sen Tissue Mapping Center (Figure 6C). Isolated islets from donors without diabetes across the lifespan underwent scRNA-Seq for transcriptomic analysis, Xenium for spatial transcriptomics, and CODEX for spatial proteomic analysis. The proportion of p21^+^ β cells in samples from donors without diabetes was 37% per scRNA-Seq (10 donors, aged 34–64 years), 15% by Xenium, and 7% by CODEX (13 donors, aged 21–71 years). These differences might reflect different sensitivity per platform as well as higher senescence induction due to the islet isolation procedure prior to scRNA-Seq.

Key β cell identity and functional genes (*INS*, *NKX6.1*) were significantly downregulated in the *CDKN1A*^+^ β cell subpopulation when compared with nonsenescent and *CDKN2A*^+^ β cells (Figure 6, D and E). To understand whether these senescent subpopulations may have originated from independent lineages or from a single population that expresses these markers in a temporally distinct fashion, as previously suggested by us and others (4, 17, 18), pseudo-time analysis of scRNA-Seq was performed in human β cells. Seven states were identified during the progression of a human nonsenescent β cell to a senescent state (Figure 6, F and G), with states 5, 6, and 7 showing upregulation of key senescent genes: *TP53*, *CDKN1A*, and *CDKN2A* (Figure 6G). State 5 expressed higher levels of *TP53*, state 6 of *CDKN1A,* and state 7 of *CDKN2A*, suggesting a temporal progression of β cells through these 3 senescent stages. In support of these being senescent states, 5, 6, and 7 had significantly higher senescence scores using the gene ontology (GO) term (Figure 6H) and senescence Fridman (Figure 6I) gene datasets.

Given the correlation between p21 with T2D and islet dysfunction in humans, spatial proteomic (CODEX) and transcriptomic (Xenium) analysis focused on CDKN1A/p21^+^ β cells from fixed whole pancreas. P21 expression was divided into quartiles (Q), with Q1 having the lowest levels and Q4 the highest. At the protein level, β cells with higher levels of P21 also had higher levels of other senescence markers: HMGB1 and XP53BP1 (Figure 6J). Consistent with their loss of identity, this same population had lower levels of key β cell proteins: INSULIN and C-PEPTIDE (Figure 6M). At the transcriptomic level, β cells with the highest levels of nuclear *CDKN1A* had the lowest levels of *INSULIN* and *MAFA*, a key functional transcription factor (Figure 6 K and L). These correlations were maintained when β cells were selected based on nuclear protein P21 levels, and their transcriptional mapping revealed lower levels of *INSULIN* and *MAFA* mRNA transcripts (Figure 6N).

These results demonstrate that a senescent and dysfunctional subpopulation of p21^+^ β cells can be identified in human islets. These cells are more abundant in tissues from individuals with T2D and thus may contribute to β cell dysfunction and T2D pathogenesis.

### Pharmacological JAK inhibition restored function of human β cells.

**Figure 7:**
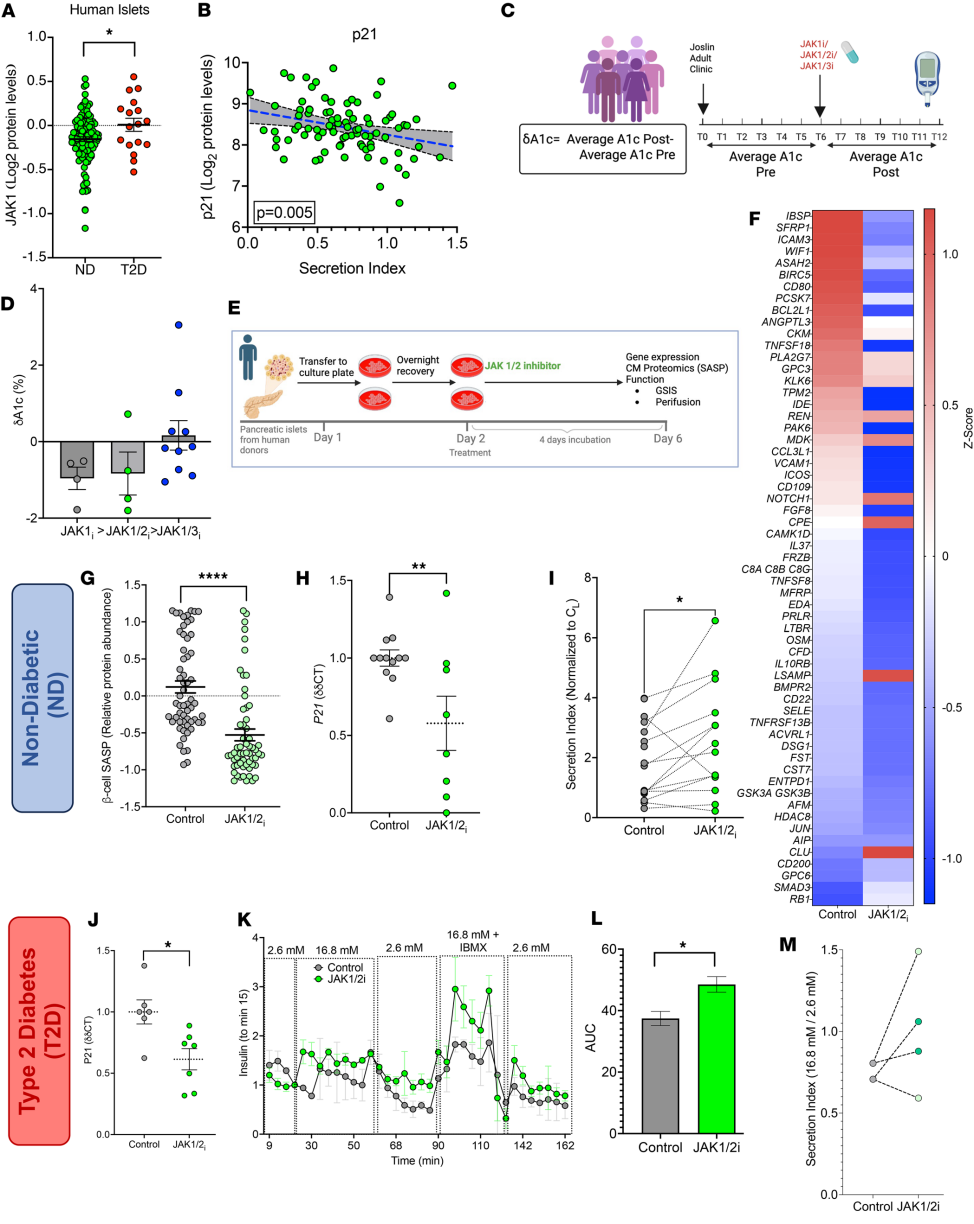
Effects of JAK1/2i on secretion of human β cell function. (**A**) JAK1 protein levels in human islets from donors without diabetes (ND) and with type 2 diabetes (T2D). (**B**) linear regression of JAK1 protein levels and secretion index from human islets. (**C**) Analysis of data from Joslin Diabetes Center’s adult clinic. Longitudinal glycated hemoglobin (A1c) levels from individuals with T2D and who had been prescribed JAKi for medical indications independent from diabetes. The effect of the JAKi treatment was measured by the change of A1c. δA1c = (average A1c post) – (average A1c pre). (**D**) Mean A1c change before and after starting treatment with a specified JAKi in people with T2D. (**E**) Workflow of human islets from ND and T2D donors treated with JAK1/2i or JAK1/3i for 4 days. (**F** and **G**) Heatmap and dot-plot of SASP (log_2_ protein abundance) secretion in conditioned media collected from human untreated (control) or treated (JAK1/2i). Top upregulated human β cell SASP proteins were selected. Paired analysis per analyte was performed between conditions. Cell numbers varied from one donor to another but were constant across treatments. Donor 1: 526,000 cells/treatment; donor 2: 192,000 cells/treatment; donor 3: 231,000 cells/treatment; donors 4 and 5: 87,500 cells/treatment. (**H**) *p21* mRNA levels in human islets from ND donors. (**I**) β cell function evaluated by GSIS in human islets from ND donors with 1–3 replicates per donor. (**J**) Effects of JAK1/2i in *p21* mRNA in islets from T2D donors. (**K**) β cell function evaluated with islet perifusion in islets from 2 donors with T2D, BMI 38.3 and 42.2, age 58 and 55 years old, respectively. (**L**) Static GSIS from 2 T2D female donors using JAK1/2i (momelotinib: dark green and baricitinib: light green), both BMI 42.2, age 49 and 55 years old. (**M**) Matched secretion index from untreated and JAK1/2i-treated islets from individuals with T2D.

To analyze the relevance of JAK inhibition in human β cells as a potential regulator of secondary senescence, data from the Human Islet Program were used (16). JAK1 protein levels were increased in islets from donors with T2D compared with islets from donors without diabetes (Figure 7A). Additionally, there was an inversely negative correlation between JAK1 protein levels and secretion index (Figure 7B), suggesting that human JAK1 might be a potential target to restore β cell function in islets with higher levels of P21^+^.

Several JAKi are approved for clinical use for different diseases (e.g., rheumatoid arthritis). Deidentified A1c levels were obtained from the Joslin Diabetes Center adult clinic from a limited number of adults with T2D who were also prescribed a JAKi (Figure 7C). Analysis of the longitudinal A1c of each patient revealed that individuals taking JAK1i and JAK1/2i had a 1% decrease in A1c levels compared with levels before the drug was prescribed (Figure 7D).

Based on this rationale, the role of JAK1/2i was tested in human islets. Islets from human donors (Supplemental Table 3) were treated in vitro with JAK1/2i for 4 days, after which CM was collected for aptamer-based proteomic analysis of the SASP (Figure 7E). The top upregulated (Figure 7F) human SASP proteins identified in human β cells (4) were compared between CM from control human cells to CM from human cells treated with JAK1/2i. Treatment with JAK1/2i resulted in a significant decrease in the secretion of SASP proteins (Figure 7, F and G), demonstrating its effectiveness in targeting human SASP.

To analyze the effects of SASP inhibition on senescent subpopulations, islets from donors without diabetes (where P21^+^ cells and a functional SASP are also found) (4) were treated as described above and analyzed for the expression of *P21* mRNA, which was decreased by JAK1/2i (Figure 7H). This was accompanied by an increase in the secretion index in islets from donors without T2D (Figure 7I). Because of the limited islet supply and the presence of senescent β cells in islets from donors of all ages, we have included a range of ages and metabolic conditions to support the pathophysiological importance of this population.

To test whether the JAK pathway was also relevant in a diseased state, islets from donors with T2D were treated with JAK1/2i for 4 days. *P21* transcription was significantly decreased (Figure 7J). From a functional perspective, JAK1/2i restored insulin secretion dynamics in the islets from 2 donors with T2D (58 and 55 years old with BMI 38 and 42, respectively) (Figure 7, K and L). This can be seen as a restoration of first-phase insulin secretion and an enhanced response to the secretagogue IBMX. Furthermore, improvement in insulin secretion was observed not only in perifusion but also in static GSIS, and similar effects were observed with 2 JAK1/2i drugs (Figure 7M).

In conclusion, the human β cell SASP is a therapeutic target to restore function during progression of T2D and in established T2D.

## Discussion

The importance of heterogeneity in cellular senescence is increasingly being recognized as a key factor in developing better-targeted interventions for various diseases. *p21* (encoded by *Cdkn1a*) and *p16* (encoded by *Cdkn2a*) are well-established markers and effectors of senescence, and their expression patterns have been used to define 3 subpopulations of β cells. We found that p21^+^ cells are the predominant senescent subpopulation of β cells in models of metabolic stress, T2D, and aging. Interestingly, the gene expression profile of these cells differed from that of p16^+^ cells: *Cdkn1a*^+^ β cells were characterized by reduced expression of hallmark genes associated with β cell identity and function, along with increased expression of commonly described SASP genes.

The coexpression of other markers of senescence and increased transcription of SASP factors support the notion that this is a bona fide senescent population rather than a population undergoing transient stress. The expression dynamics observed in islets from mice treated with S961 for 2 weeks suggested that *Cdkn1a* could be an initial driver of β cell senescence, and *Cdkn2a* would increase at a later stage, a process that has also been previously proposed by us and others (4, 18). This concept was further supported by pseudo-time analysis of mouse and human scRNA-Seq of β cells, by *Cdkn2a*^+^ cells distinctly separating from *Cdkn1a*^+^ and the nonsenescent cells in the PCA plot, and by the conditional knockdown *of p21* in mouse islets. Even though these populations are temporally asynchronous, their specific control should be studied further.

Our data support the induction of secondary senescence in β cells by *Cdkn1a*^+^ cells. This effect was shown with full SASP in CM, with a subset of selected SASP factors and by each factor individually. In these different conditions, naive cells exposed to SASP exhibited increased expression of senescence genes (particularly *p21*) and became dysfunctional, consistent with secondary senescence being an important effector of β cell dysfunction in models of insulin resistance and progression to T2D. The role of the β cell SASP as an inducer of secondary senescence underscores the importance of therapeutically targeting the secretome of senescent cells through pharmacological interventions. Treatment of β cells with JAK1/2i decreased the expression of senescence markers and improved cell function in models of early T2D. JAK1/2i compete with JAK1 and 2 to reduce the intracellular signaling of cytokines and growth factors, thereby mitigating their transcriptional effects (19, 20). In human β cells, JAKi significantly decreased secretion of SASP factors and, based on RNA-Seq data, it also significantly decreased signaling by interleukins (IL-2, IL-6, IL-9, IL-20, IL-21, IL-27, IL-28, IL-35), IFN-γ, and TP53 activity in mice. Similar inhibition by JAKi has been reported in the adipose tissue (21). The specific roles of each of these pathways in restoring the function and identity of β cells will be the subject of future studies.

If JAKi are considered as an additional strategy to restore β cell function during progression to T2D, several points deserve reflection given their multiple downstream effects. The JAK/STAT pathway plays a dual role in β cells, underscoring the need for selective modulation. Prolactin-induced JAK2/STAT5 activation promotes β cell survival by increasing the expression of antiapoptotic protein BCL-XL and protects against glucolipotoxicity-induced cell death in rodent and human β cells (22, 23). This suggests that enhancing JAK2/STAT5 signaling may maintain β cell mass, but therapeutic strategies must avoid excessive activation that could promote inflammation.

Additionally, the JAK/STAT pathway mediates proinflammatory effects in response to extracellular cytokines and chemokines. The IL-6/JAK2/STAT3 pathway has been shown to promote inflammation and ER stress in diabetic β cells (24). Conceptually, it cannot be ruled out that JAKi in our models modulate inflammation, given its known effects on the immune system. Consistent with the effects of JAK signaling on the immune system, the main reported side effects of JAKi are infections, particularly viral infections, which can be mitigated with vaccination (25). Increased risk of cardiovascular and thromboembolic events are reported side effects — these could be avoided by specifically targeting these drugs to β cells through chemical manipulation. Also, some of these effects might be dose dependent.

Supporting the use of JAKi to protect human β cells, daily administration of JAK1/2i in low doses to people, including children, with recent onset type 1 diabetes preserved β cell function (26) and was well tolerated.

In conclusion, senescent β cell heterogeneity provides the biological basis for differentially targeting specific subpopulations and counteracting their effects on neighboring cells during diabetes progression. Considering that multiple pharmacological targeting strategies are available, SASP inhibition with JAKi was shown to restore human β cell function and enhance cell survival in models of early β cell dysfunction. These concepts will further our understanding of senescence biology and support the rational development of potentially novel therapeutic strategies for diabetes.

## Methods

### Sex as a biological variable

Our study examined male and female animals and human samples. Similar findings are reported for both sexes.

### Animals

Male *C57BL/6J* mice (8-week-old or 6–9-month-old-retired breeders) obtained from The Jackson Laboratory (catalog 000664) were used for islet isolation and CM generation. Male mice were chosen at this stage because they are more susceptible to develop insulin resistance after dietary or pharmacological interventions. All experiments were performed in the animal facilities at the Joslin Diabetes Center with approval from its IACUC. Mice were kept on a 12-hour light/12-hour dark cycle with water and food provided ad libitum. A housing temperature of 22.2°C–22.7°C was maintained.

### p21-tdTom mice

To generate a mouse in which tdTomato expression was driven by the *p21* gene, the P2A-tdTomato transgene was inserted in place of the stop codon at the 3′ end of the coding region. The Easi-CRISPR design methodology was followed as previously published (27, 28). Bioinformatic analysis of the gene for gRNA identification was performed by crispor.tefor.net. Microinjection was performed into embryos from 4-week-old *C57 Bl6* female donors (The Jackson Laboratory, catalog 000664), which were subsequently implanted into 6-week-old Swiss Webster females (Charles River Laboratories, catalog Crl:CFW(SW)). Junction sequencing was performed to verify the construct.

The GTT showed no significant differences among p21-tomato reporter versus non-reporter mice for both sexes in terms of glucose levels (Supplemental Figure 5, A and B).

### Cell lines

Murine MIN6 cells were originally provided by Jun-ichi Miyazaki from the University of Osaka Graduate School of Medicine, Osaka, Japan, and the sex of the cell line was not available. The cell line was maintained in DMEM (high glucose, with 4,500 mg/L glucose, L-glutamine, sodium pyruvate, and sodium bicarbonate) from Sigma-Aldrich supplemented with 15% FBS from Summerlin Scientific, 1% penicillin-streptomycin (5,000 U/mL) from Gibco, and 0.05% β-mercaptoethanol (99% cell culture tested) from Sigma-Aldrich. The cells were incubated at 37°C with 5% CO_2_.

### Mouse primary islets

*p21-tdTomato* and *C57BL/6N* mice were used for islet isolation. Isolation was performed according to the protocol described by Gotoh et al. (29). The islets were then hand-picked under a StereoZoom 7 microscope and plated in petri dishes with islet media. The media were composed of RPMI 1640 (1×, [+] L-glutamine) (Gibco) supplemented with 10% FBS (Summerlin Scientific) and 1% penicillin-streptomycin (5,000 U/mL; Gibco). For the dispersion of islets, we used TrypLE Express Enzyme (1×, [–] phenol red) (Gibco). The islets were incubated at 37°C with 5% CO_2_.

### Human primary islets

Human islets were obtained from donors through the Integrated Islet Distribution Program (IIDP) and from Prodo Laboratories, Inc. (Supplemental Table 3). Immediately upon arrival, the islets were cultured in CMRL medium 1066 (1×, [–] glutamine) from Gibco containing 10% FBS (Summerlin Scientific) and 1% penicillin-streptomycin (5,000 U/mL) (Gibco). For the dispersion of islets, we used TrypLE Express Enzyme (1×, [–] phenol red) (Gibco). The islets were incubated at 37°C with 5% CO_2_.

### Whole pancreas preparation

Whole pancreata were obtained from the University of Texas Health San Antonio from IRB-exempt brain-dead organ donors and shipped to Joslin Diabetes Center under approved protocols. Donors were screened by the Texas Organ Sharing Alliance according to established criteria, including age 20–60 years or older, and excluded for conditions such as type 2 diabetes, pancreatic malignancy, pancreatitis, use of agents with senolytic activity, or infection. Pancreata were sectioned into head, body, tail, and regions along the pancreatic margin, with samples processed as both FFPE and frozen tissues for downstream histological and molecular analyses. Tissue preservation and shipping procedures were standardized to ensure sample integrity prior to analysis at Joslin Diabetes Center.

### JAKi treatment in vitro

The following drug concentrations were used for treatment and were diluted in CMRL medium 1066 1× (See Human Primary Islets): 1 μM JAK1/2i (momelotinib) and 100 nM JAK1/2i (baricitinib). The concentration of momelotinib was based on published data (21), which showed effective decreases in the transcription of specific SASP factors. Human and mouse cells were incubated for 4 days in wells previously treated with PEI and Geltrex.

### JAKi treatment in vivo

After 2 weeks of HFD feeding (60% v/v), the animals (8-week-old male *C57BL/6N* mice) were subdivided into 3 groups: saline, JAK1/2i momelotinib, vehicle (HFD, 0.9% NaCl), or JAK1/2i (HFD + momelotinib, 12.5 mg/kg/day) administered through an osmotic pump in 8-week-old animals. All control animals received vehicle via oral administration (NaCl 0.9%). Blood glucose levels in the mice in all groups were monitored biweekly, and the mice were euthanized after treatment (4 weeks for HFD). The pancreatic islets were then isolated for qPCR analysis or bulk RNA-Seq. Heart blood was collected through cardiac puncture to measure circulating insulin in the mice.

### GTT

GTTs (i.p.) were performed before and after senolytic or senomorphic treatment, as well as in p21-tdTom–positive and –negative littermate mice. After a 6-hour fast, mice received i.p. glucose (2 g/kg body weight), and blood glucose levels were measured at baseline and multiple time points up to 120 minutes after injection using a glucometer. Blood samples collected at 0 and 15 minutes were additionally used for serum insulin quantification by ELISA.

### Generation of SASP-CM

MIN6 cells were cultured in T175 flasks and treated with either 50 μM bleomycin or DMSO control for 48 hours to induce senescence. After treatment, cells were recovered in normal medium for 72–96 hours and subsequently incubated in serum-free medium for 24 hours to generate CM. The collected media were used in downstream experiments as BCM or control CM.

### Conditional knockdown with siRNA

MIN6 cells were transfected with Opti-MEM–based siRNA (50 nM) mixtures using Lipofectamine RNAiMAX and incubated for 48 hours before downstream analyses. For primary mouse islet experiments, dispersed islet cells were cultured overnight and transfected with scrambled or p21-targeting siRNA using similar conditions. After transfection, media were replaced and cells maintained for 4, 6, 8, 10, and 12 days after transfection. Functional assessment included GSIS assays, and RNA was isolated from harvested cells for reverse transcription and qPCR analysis of gene expression.

### In vitro recombinant protein treatment

Approximately 300,000 MIN-6 cells were plated into 24-well plates with 500 μL of DMEM supplemented with 15% FBS. The cells were incubated at 37°C with 5% CO_2_ for 24 hours to allow attachment. The concentrations of the following recombinant proteins were compared with those detected in plasma by immunoassay (https://www.proteinatlas.org): DUSP3 (1.3 μg/L), GDF15 (1.3 μg/L), IDE (0.31 μg/L), LSAMP (150 μg/L), HSP90 (71 μg/L), and GSTP1 (69 μg/L). The recombinant proteins were added to RPMI 1640 media without FBS supplemented with 1% BSA. Details are provided in Supplemental Table 2. The cells were treated for 48 hours, followed by incubation for 48 hours in normal media. After treatment, the cells were subjected to qPCR.

### GSIS in vitro

Static GSIS assays were performed using Krebs-Ringer HEPES buffer containing defined concentrations of salts, glucose, and BSA. Islets and/or FACS-sorted cells from P21-tdTom mice were preincubated in low-glucose conditions, followed by sequential low- and high-glucose incubations. Supernatants and corresponding cells were collected after each step for normalization to RNA or DNA content and stored for downstream analyses. Insulin secretion was quantified using ELISA or ultrasensitive homogeneous time resolved fluorescence insulin assays. The raw insulin secretion values at low and high glucose are summarized in Supplemental Table 7.

### Real-time qPCR

RNA extraction was performed using the RNeasy Micro kit, followed by reverse transcription of RNA to cDNA using SuperScript IV Reverse Transcriptase. Gene expression was quantified by qPCR using PowerUp SYBR Green Master Mix, with β-actin used for normalization. Primer sequences used in the study are provided in Supplemental Table 4.

### Immunostaining, image acquisition, and morphometric analysis

The cells were fixed with 10% formalin and permeabilized with 0.3% Triton X-100 (Alfa Aesar). The cells were blocked with normal donkey serum at a 1:50 dilution in PBS. The sections were incubated overnight at 4°C with anti-Ki67 antibody. The next day, they were incubated with anti-insulin and then with fluorochrome-conjugated secondary antibodies and mounted with Fluoroshield with DAPI (Sigma-Aldrich).

The antibodies used are listed in Supplemental Table 5. For each stain, all images were taken with the same settings in confocal mode using a Zeiss LSM 710 microscope such that comparisons across conditions could be made.

The intensity for proliferation analysis and the area for cell density were quantified using ImageJ (NIH).

Quantification of fluorescence intensity was performed with Fiji, an open source distribution of ImageJ. TdTOMATO fluorescence was quantified, followed by classification into higher or lower signaling categories based on the mean integrated density. Lower signaling corresponded to values in the lower half of the distribution, whereas higher signaling represented values in the upper half. To address islet heterogeneity, immunostaining was adjusted using the corresponding tdTomato integrated density values. This method facilitated the comparison of the impact of increasing tdTOMATO intensity and, consequently, P21 expression and other marker β cell markers: INSULIN, MAFA, NKX6, and PDX1. Staining details are provided in the Supplemental Data.

### Bulk RNA-Seq

Islet isolation was performed according to the protocol described by Gotoh et al. (29). RNA was extracted from isolated islets using an RNeasy Micro kit and sent for sequencing to DNA Link. Data analysis was performed by the Bioinformatics and Biostatistics Core at Joslin Diabetes Center.

### scRNA-Seq: mouse islets

Our previously published (4) scRNA-Seq database in NCBI’s Gene Expression Omnibus (GEO GSE149984) was reanalyzed with β cells clustered into subpopulations using the expression of *Cdkn1a* and *Cdkn2a*. Briefly, ALZET mini-pumps with the insulin receptor antagonist S961 or PBS were surgically inserted into mice for 2 weeks as described by Midha et al. (4). Three groups of mice (*n* = 4 animals per group) were used: control (PBS pumps), S961 (20 nmol/L/week for 2 weeks), and recovered (mice with S961 removed for 2 weeks). For scRNA-Seq, islets were isolated from all mice in each group on the same day, cultured overnight, and dispersed. There were 2,939 β cells in the control group, 2,896 cells in the S961 group, and 2,513 cells in the recovery group.

For βSenMayo, the list of genes described in Saul et al. (10) was assessed in different senescent subpopulations of β cells from the control condition. Only those genes with quantifiable gene reads in all 3 subpopulations were included in the analysis to exclude those with extremely low expression in β cells.

A detailed description of the bioinformatic analysis of the scRNA-Seq data can be found in the Supplemental Methods. The scRNA-Seq data are in NCBI’s GEO (GSE149984).

### scRNA-Seq, CODEX, and Xenium of human islets

A full description is provided in the Supplemental Data.

### β-Gal activity assay

To measure senescence-associated β-gal activity, we used the Dojindo Cell Count Normalization kit (C544) combined with the Cellular Senescence Plate assay kit - Spider-βGal (SG05) following the manufacturer’s protocol for combined analysis.

### Flow cytometry

#### β-Gal^–^ and β-gal^+^ human and mouse cells.

Human and mouse β cells were sorted using flow cytometry based on β-gal activity to divide them into senescent (β-gal^+^) and nonsenescent (β-gal^–^) populations. β-Gal activity was measured using an Enzo cellular senescence live-cell senescence assay kit (ENZ-KIT130-0010) following the manufacturer’s instructions while optimizing the substrate incubation time to 1 hour. Sorting was performed using a DakoCytomation MoFlo cytometer or FACSAria in the Joslin Diabetes Research Core Flow Cytometry Core. Primary islets were incubated with antibodies against CD45 and CD11b to eliminate immune cells.

#### p21-tdTom mice.

Islets from *p21-*tdTom mice were isolated, dispersed into single cells, and sorted using a MoFlo cytometer based on Tomato red fluorescence. The cells were collected in media, plated in 96-well plates overnight, and assessed for GSIS the following day. The results were normalized to the DNA quantity. A detailed protocol for staining for *p16^Ink4+^* in FACS is provided in the Supplemental Methods and Supplemental Table 6. The gating criteria are shown in Supplemental Figure 7, and 90% enrichment of β cells was achieved, as previously published (3).

### Proteomics of human CM from β-gal–/β-gal+ human cells and control and JAK1/2i-treated islet cells

After sorting, β-gal^+^ and β-gal^–^ cells were plated, and CM was generated to compare the SASP secreted by either cell type. CM from control and pharmacologically treated cells was generated as described above. The generated CM was analyzed using SomaScan proteomics at the Beth Israel Deaconess Medical Center Genomics Proteomics Core. Proteomic data were normalized, log_2_-transformed, and analyzed by PCA to assess differences among samples. Differentially secreted proteins were identified using Limma, and statistical significance was determined using moderated paired 2-tailed *t* tests.

### Statistics

The data are shown as mean ± SEM, and *P* values less than 0.05 were considered significant. For statistical analysis, unpaired or paired 2-tailed Student’s *t* tests and 2-way ANOVA were used to compare groups. Normality and log normality analyses were performed, and nonparametric statistics (Kruskal-Wallis test, Mann-Whitney *U* test, and Wilcoxon’s test) were performed when samples did not meet the criteria for a normal distribution. GraphPad Prism software was used for graphs and statistical analysis (significance and distribution). Data outliers were determined using the Grubbs outlier test or a deviation of more than 2 SDs from the mean.

### Study approval

All experiments were conducted at Joslin Diabetes Center with approval of its IACUC under protocol 063-2024. Studies involving human pancreas were determined “Not Human Research” (STUDY 00000152) by Joslin’s Committee on Human Studies based on lack of access to any identifying information and specimen collection at other institutions.

### Data availability

Human proteomic β-gal data are available in the NCBI’s GEO (GSE150285), and human data for the control and JAK1/2i datasets are available in GEO (GSE162521). Also, mouse S961-treated scRNA-Seq data are available in GEO (GSE149984) and bulk RNA-Seq HFD with or without JAKi data are available in GEO (GSE294866). Human scRNA-Seq, CODEX (phenocycler), Xenium, and H&E raw datasets are publicly available via the SenNet Consortium portal (https://data.sennetconsortium.org/). Code associated with processing and analysis will be made available via the SenNet GitHub page (https://github.com/sennetconsortium).

Supporting data values associated with the main manuscript and supplemental material, including values for all data points shown in graphs and values behind any reported means, are provided as Supporting Data Values Excel files.

## Author contributions

CA, PC, KI, FH, SP, SS, SL, and MJ acquired, analyzed, and interpreted the data and wrote, edited, and revised the manuscript. CC and JAD acquired the data. DB, SD, and JHC analyzed the data. AP did experimental and data analysis. AM generated the *p21-*tdTomato mice with the assistance of the mouse genomic core at Joslin Diabetes Center, and HP analyzed the proteomic, scRNA-Seq, and RNA-Seq data. CAM designed the project; acquired, analyzed, and interpreted the data; and wrote, edited, and revised the manuscript. JLW contributed to organ retrieval, scRNA-Seq, CODEX, and Xenium on whole human pancreas and human islets. SE, FGC, JHC, VDG, JLK, TT, NM, GAK, and PR designed the experiments, wrote, edited, and revised the manuscript. All authors read, revised, and approved the manuscript. The following are SenNet KAPP-Sen members: JAD, DB, SD, SE, AP, FGC, JHC, GAK, NM, PR, TT, JLK, and VDG.

## Conflict of interest

Part of the work in this manuscript has been submitted for a patent JDP-216 on January 16, 2024, as a US provisional patent, 63,621,239.

## Funding support

This work is the result of NIH funding, in whole or in part, and is subject to the NIH Public Access Policy. Through acceptance of this federal funding, the NIH has been given a right to make the work publicly available in PubMed Central.

- NIH/National Institute on Aging grant U54AG075941 SenNet Initiative KAPP-Sen Tissue Mapping Center (to multiple prinicpal investigators: GAK, NM, PR, and VDG.)
- NIH grants 1R01DK132535 (to CAM), P30DK036836 (to the Joslin Diabetes Research Center), 1R25DK113652 (to SP), R25 DK140752 (to SL), R37 AG13925 (to JLK and TT), and 2UC4DK098085 (to IIDP).
- Beatson Foundation grant 2020-010 (to CAM).
- Richard and Susan Smith Family Foundation Award (to CAM).
- Institutional Startup Funds (to CAM).
- Hevolution grant HF-GRO-23-1199148-3 (to JLK and TT).
- Connor Fund (to JLK and TT).
- Robert and Theresa Ryan Foundation (to JLK and TT).
- Noaber Foundation grants (to JLK and TT).
- The Mampei Suzuki Diabetes Foundation (to KI).
- Mary K. Iacocca Fellowship (to KI).

## Supplementary Material

Supplemental data

Supporting data values

## Figures and Tables

**Table 1:**
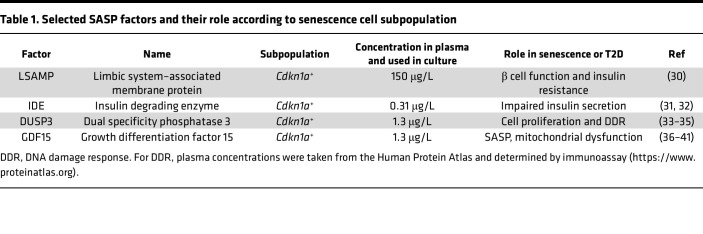
Selected SASP factors and their role according to senescence cell subpopulation

## Version 1 06/09/2026

In-Press Preview

## Version 2 07/22/2026

Electronic publication
