# Supplementary material for: p21-senescent cells drive pancreatic islet dysfunction through targetable paracrine signaling in type 2 diabetes
Source: JCI Insight. 2026 Jun 9;11(14):e197310. doi: 10.1172/jci.insight.197310 (PMC13461164; doi:10.1172/jci.insight.197310)
Supplement: Supplementary file 1 [file jciinsight-11-197310-s215.pdf]

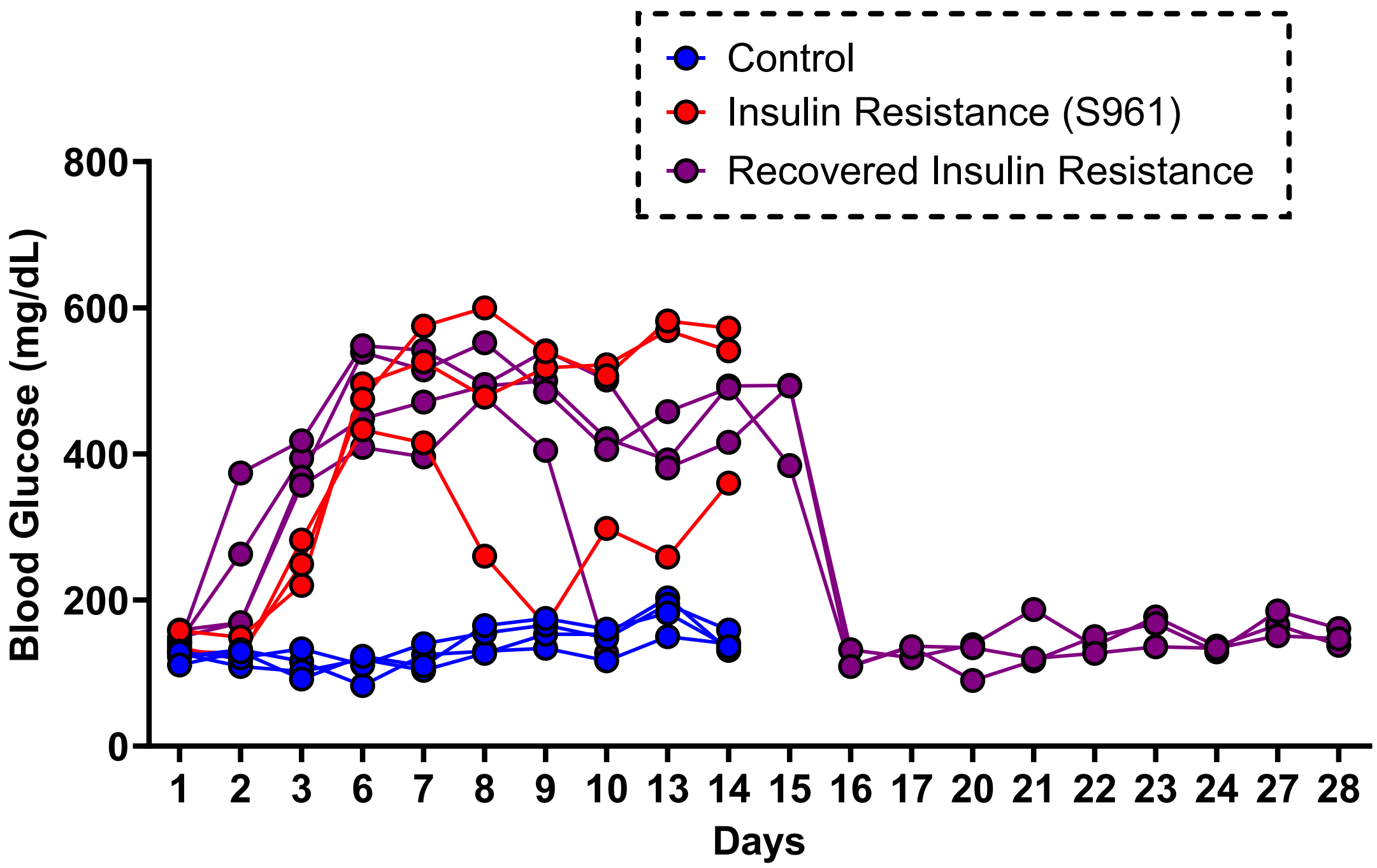

Suppl. Fig. 1 related to Fig. 1A (**A**) Blood glucose levels in mice treated with S961 with and without 2-week recovery period. These data have been previously published in (6)

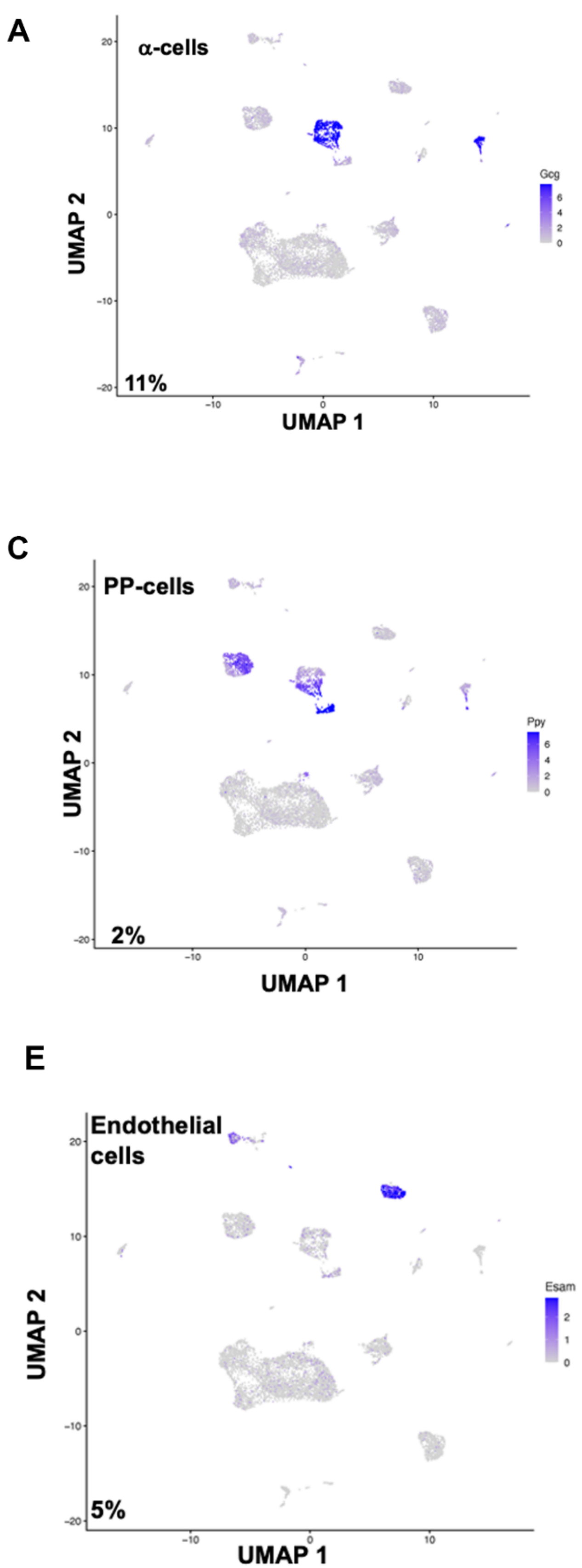

Suppl. Fig. 2 related to Fig. 1B. UMAP of non-β-cell subpopulations after scRNASeq as determined by signature genes in mice. (**A**) Alpha cells by *Gcg* expression; (**B**) Delta cells by *Sst* expression; (**C**) PP cells by *Ppy* expression; (**D**) ductal cells by *Krt19* expression and (**E**) endothelial cells by *Esam* expression.

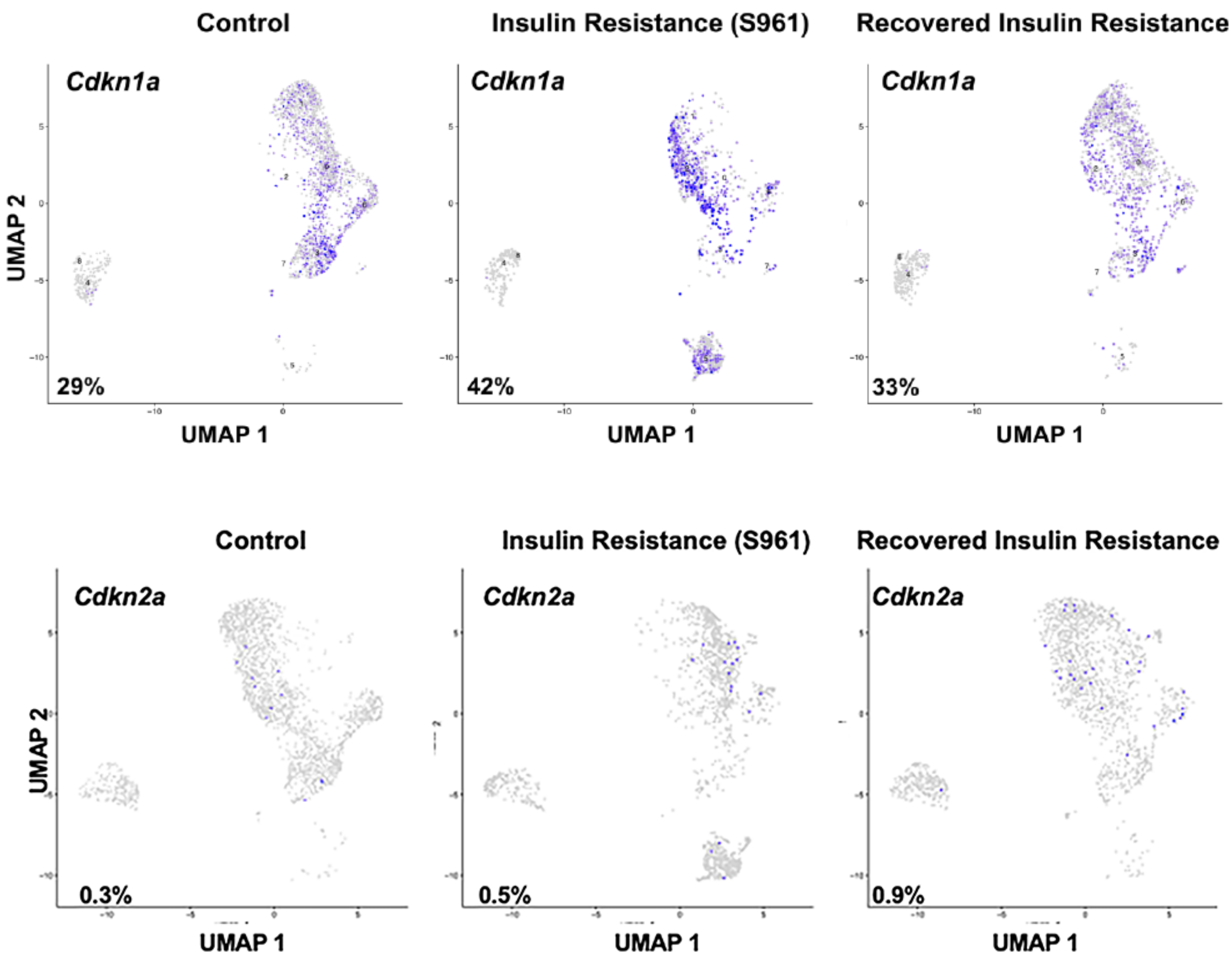

Suppl. Fig. 3 related to Fig. 1B. UMAPs showing the percentage of *Cdkn1a*$^+$ β-cells under the three different metabolic conditions. UMAP showing the percentage of *Cdkn2a*$^+$ β-cells under three different metabolic conditions as determined by scRNASeq in 8-9 month-old C57Bl/6J mice. Purple color intensity is proportional to magnitude of gene expression.

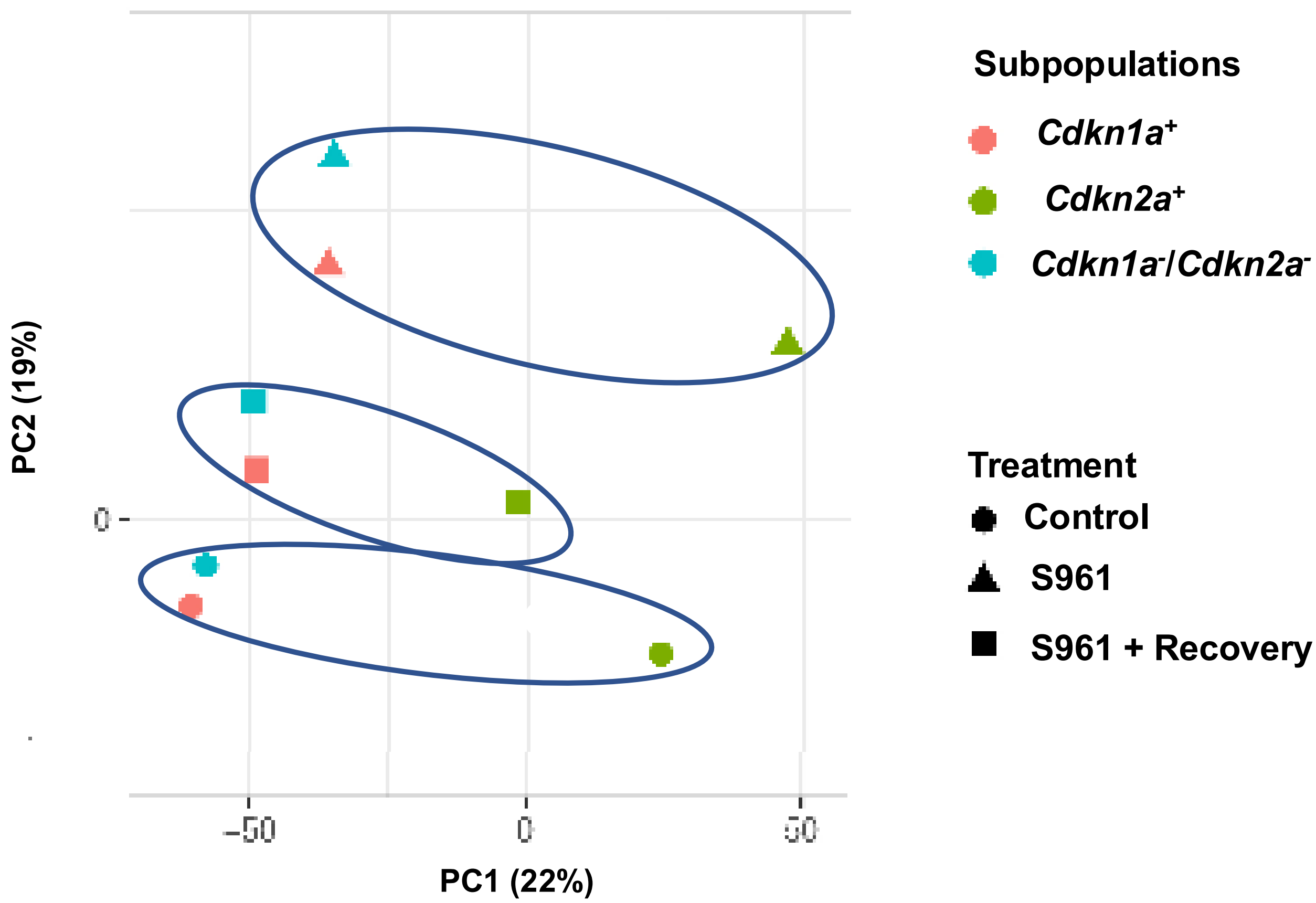

Suppl. Fig. 4 related to Fig. 1F-I. PCA analysis of the z-scores of mouse β-cells shown in Figs. 1E, F shows significant clustering by treatment, Therefore, results are shown for all three treatments in the 3 cell subpopulations.

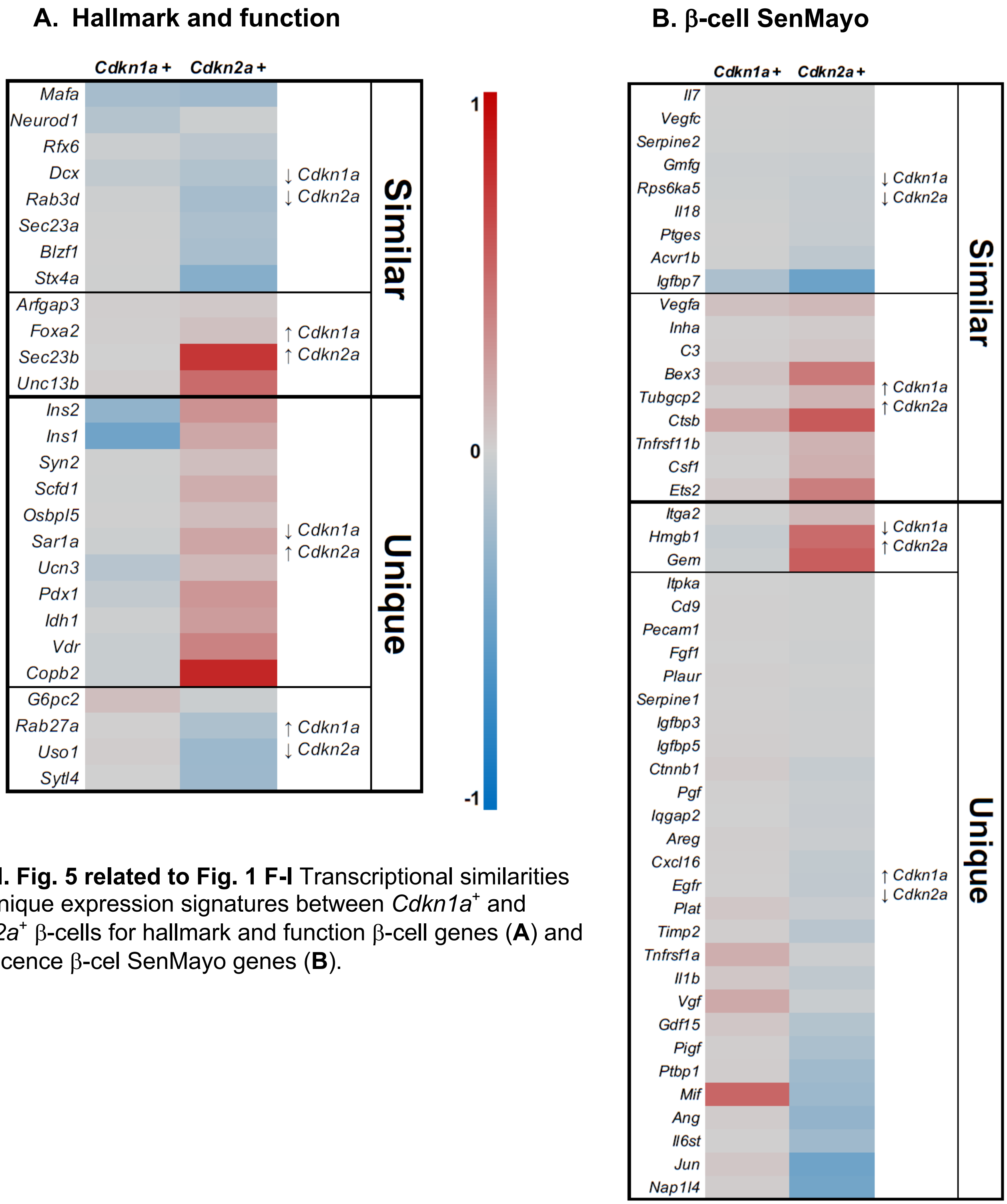

Suppl. Fig. 5 related to Fig. 1 F-I Transcriptional similarities and unique expression signatures between *Cdkn1a*+ and *Cdkn2a*+ β-cells for hallmark and function β-cell genes (**A**) and senescence β-cel SenMayo genes (**B**).

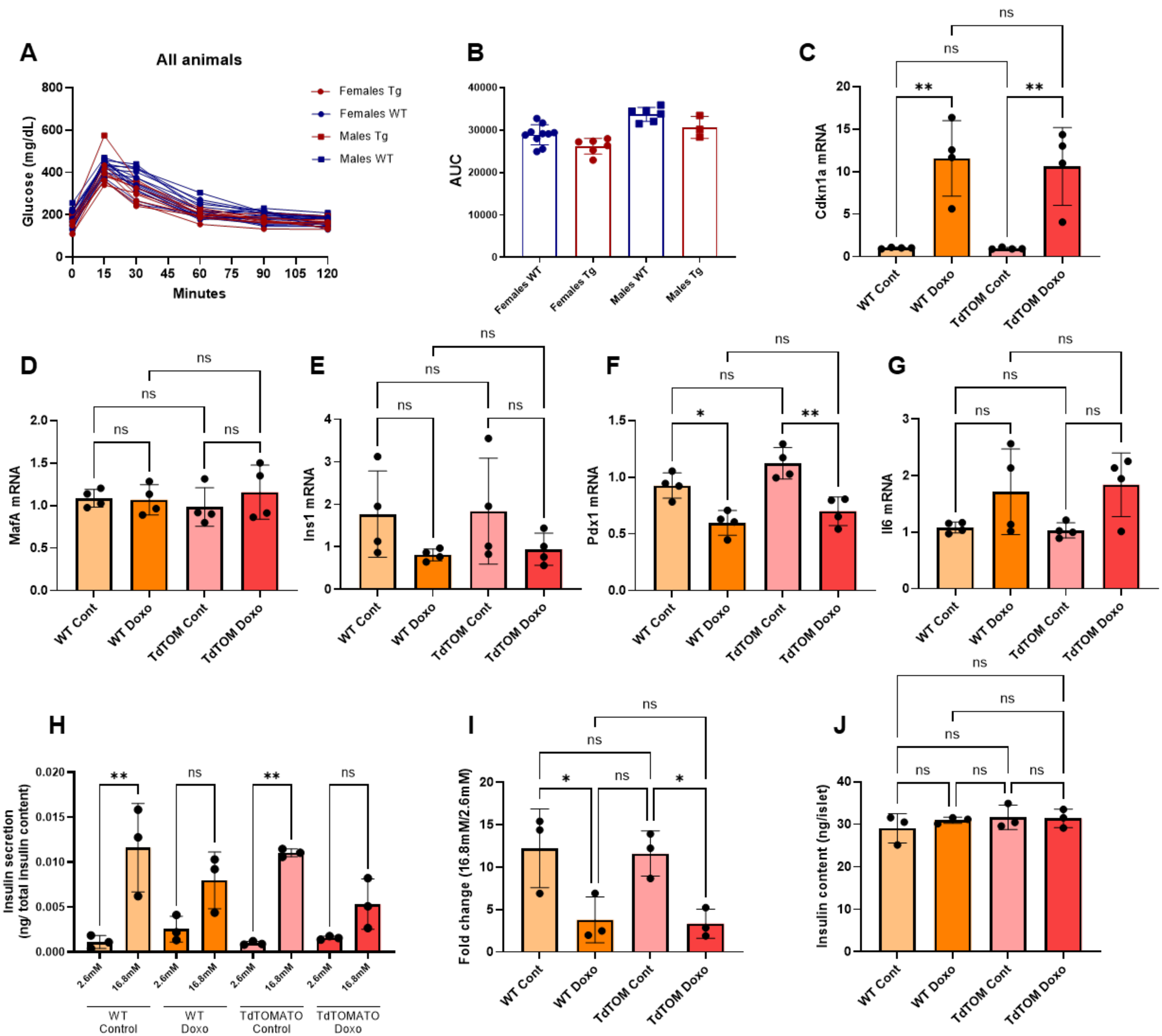

Suppl. Fig. 6 related to Fig. 2A. p21-tdTom transcript effect on glucose metabolism and islet gene expression and function. (A, B) IPGTT and area under the curve of glucose from p21-tomato reporter mouse versus wild type (WT) mouse. (C-G) The genes expression levels from islets treated with/without doxorubicin in both tdTomato and WT mice. n=4 for each group; *p<0.05, **p<0.01 by one-way ANOVA. (H-J) Static GSIS of the islets from tdTomato and WT treated with doxorubicin, and the fold change of insulin secretion from static GSIS shown in I. Corresponded islets were collected and measured insulin content in J. n=3 for each group; *p<0.05, **p<0.01 by one-way ANOVA.

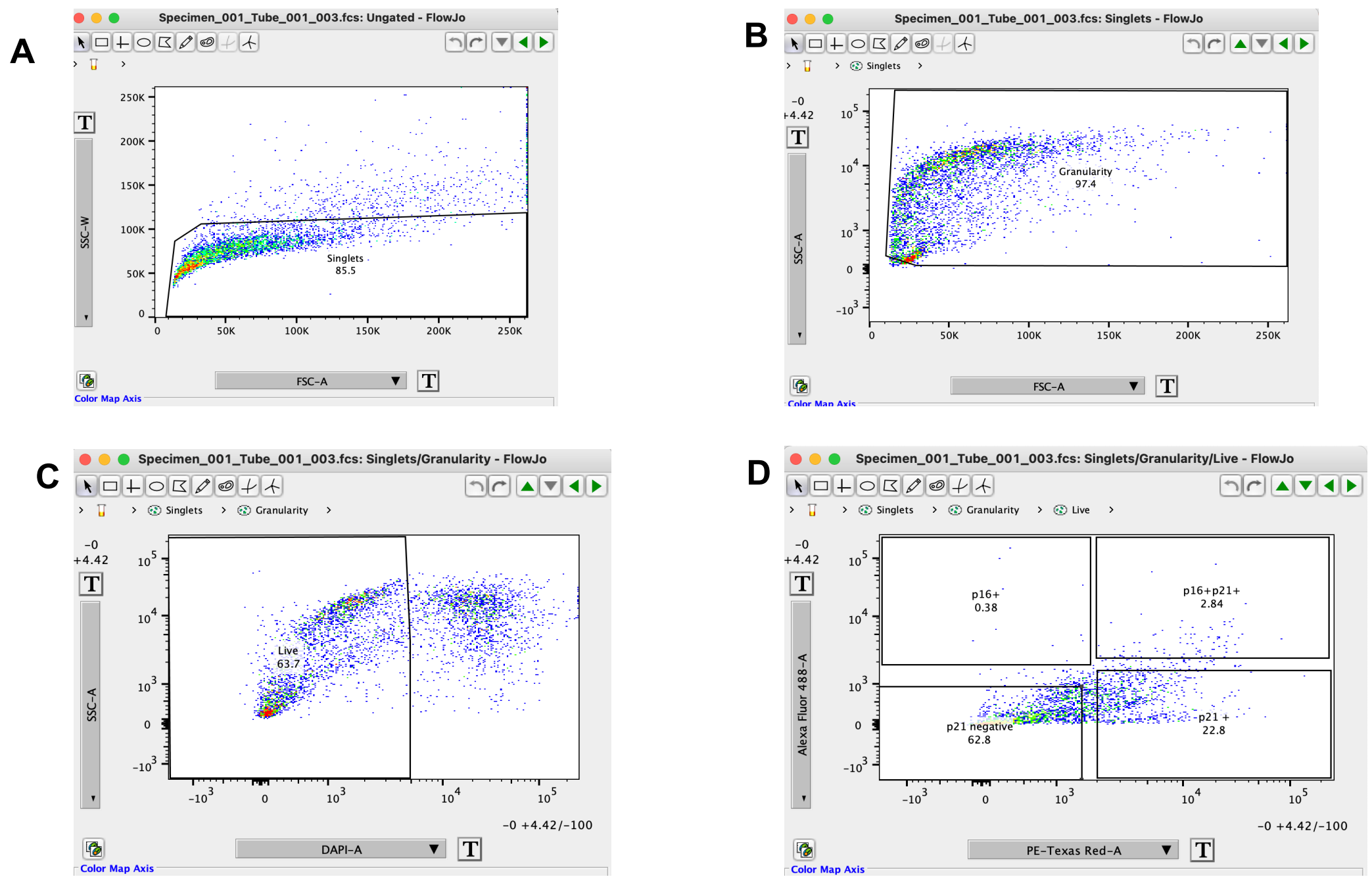

Suppl. Fig. 7 related to Fig. 2D, 3L and 5C. Flow cytometry gating strategy for identification of senescent subpopulations. (**A**) Gating to identify single cells followed by (**B**) Gating based on granularity, (**C**) Gating to identify live cells through the exclusion of DAPI and (**D**) Identification of 3 cell subpopulations based on the intensity of dTomRed for P21 and Alexa fluor-488 for P16.

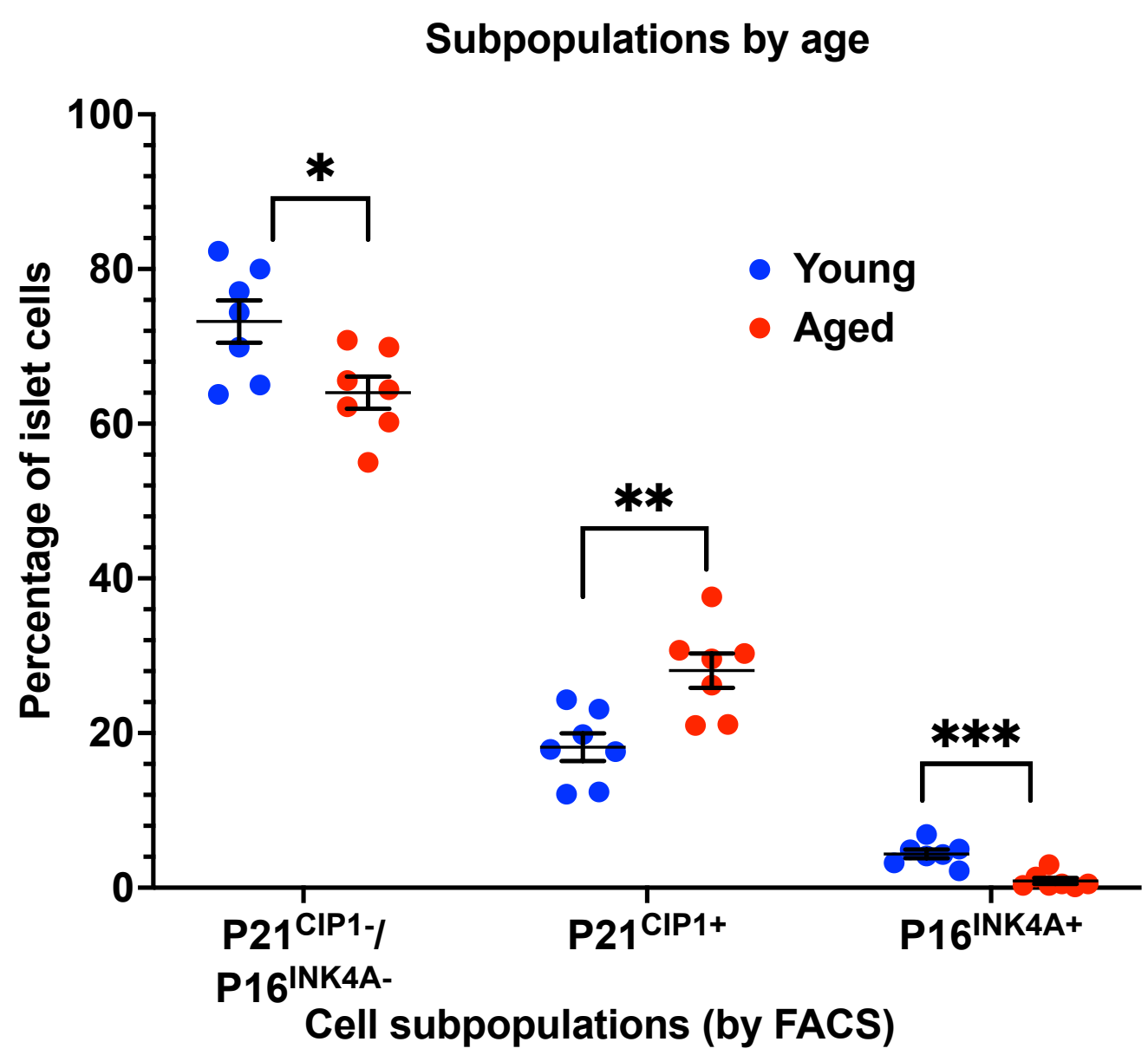

Suppl. Fig. 8 related to Fig. 2E. Percentage of p21$^+$ (encoded by *Cdkn1a*), p16$^+$ (encoded by *Cdkn2a*) and double *P21*$^-$/ *P16*$^-$ islet cells by flow cytometry from islets isolated from young (3 months) and aged (18 months) *p21*-tdTomato mice and stained for *p16*, n=7 mice in both age groups.

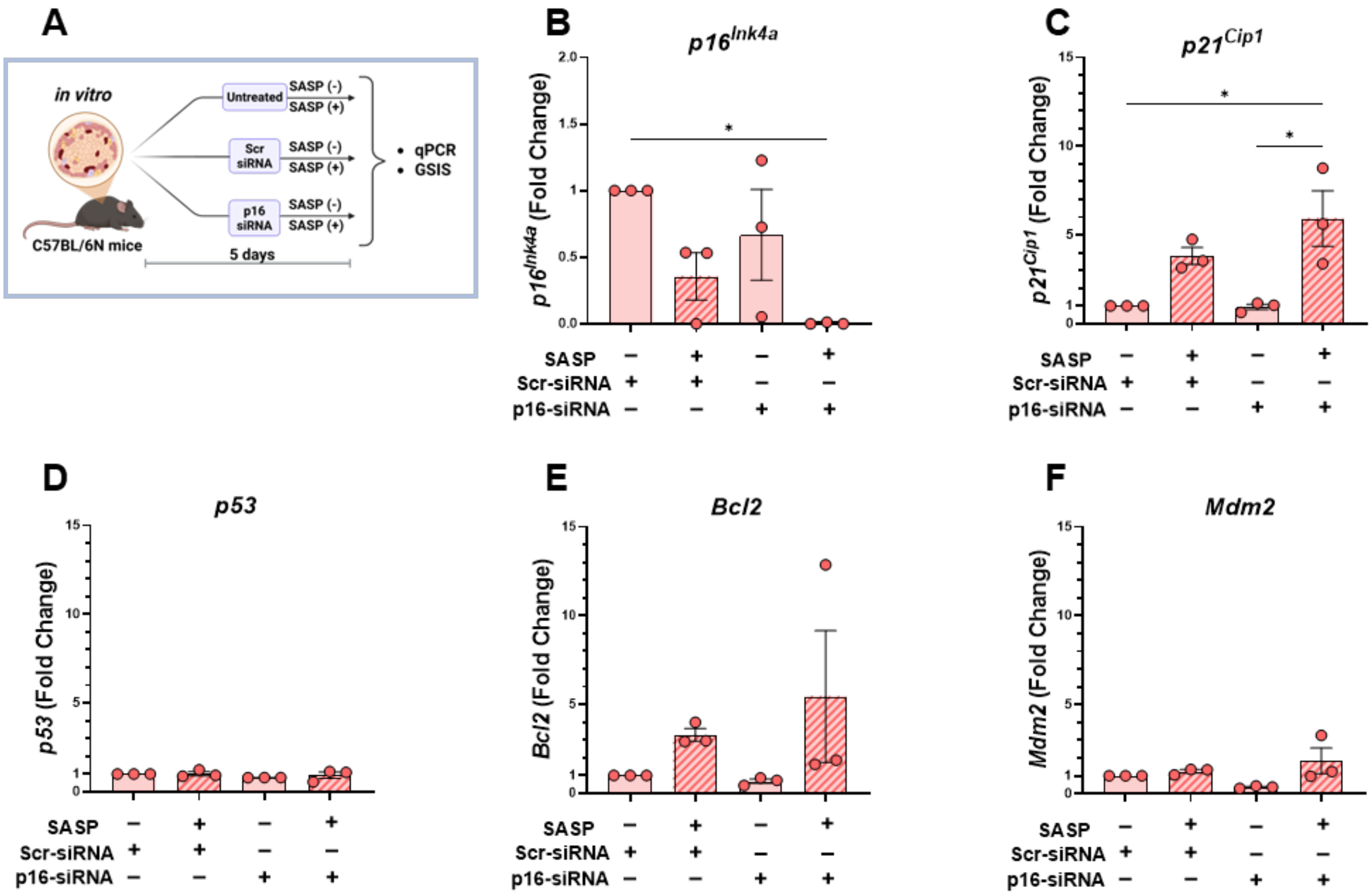

Suppl. Fig. 9 related to Fig. 4E. Treatment of islets from C57BL/6N mice with Cdkn1a SASP factors (LSAMP+DUSP3+GDF15+IDE) during 5 days after p16-siRNA knockdown. (**A**) Experimental design. (**B-F**) Senescence-related genes. *p<0.05. one-way ANOVA, Mean+-SEM.

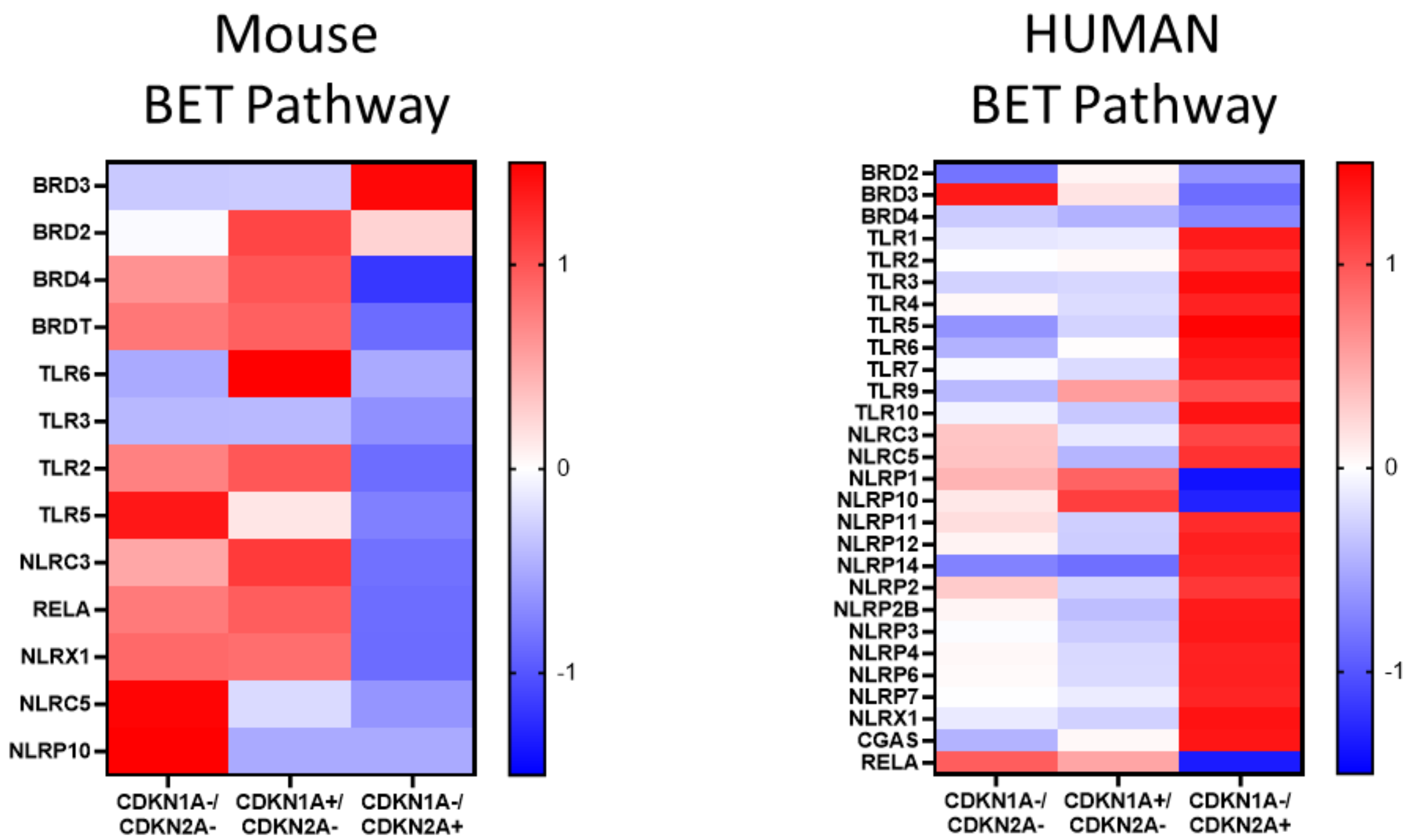

Suppl. Fig. 10. Related to Fig. 5A The heatmaps of the BET pathway and its downstream gene levels in scRNASeq data from human and mouse islets.

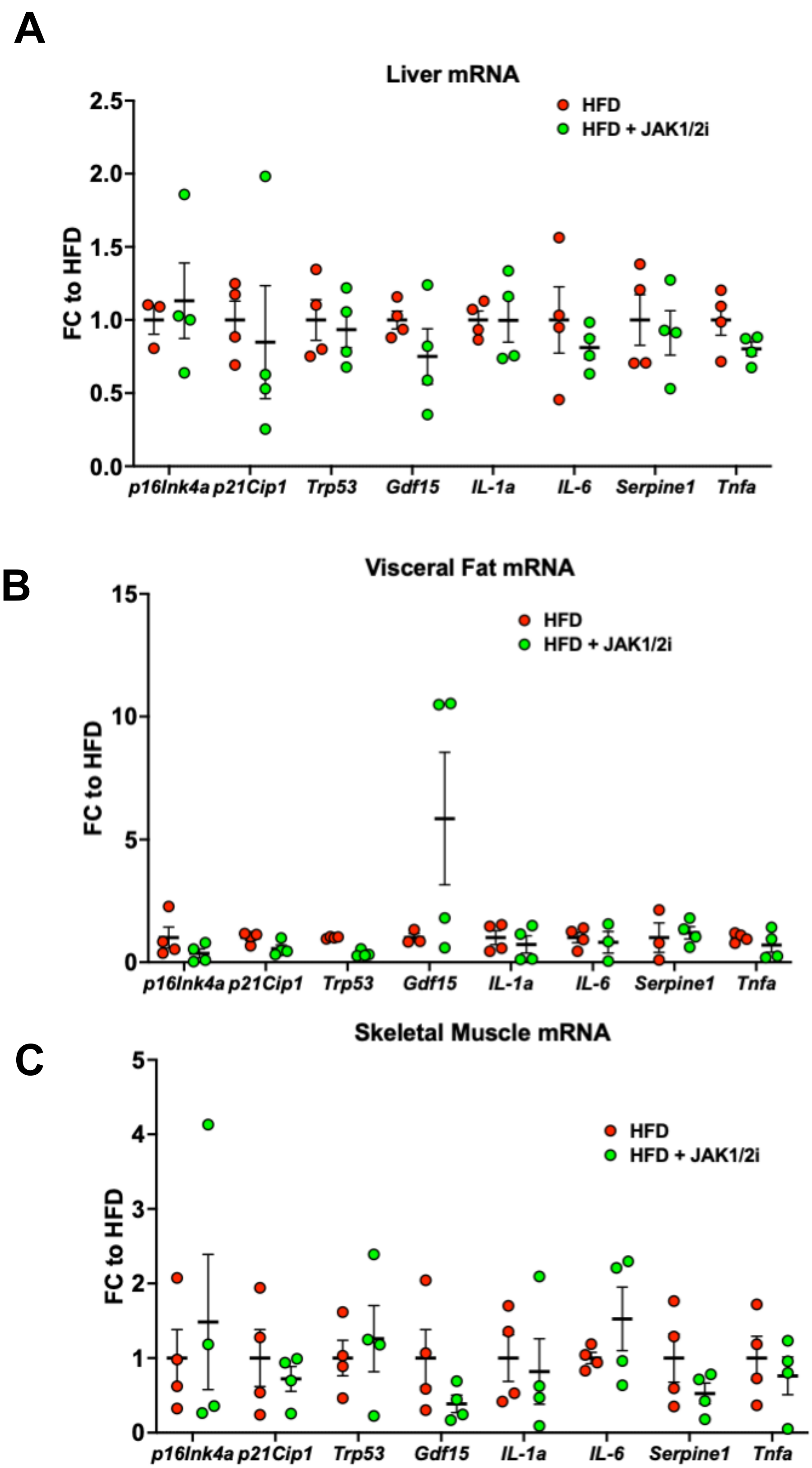

Suppl. Fig. 11 related to Fig. 5. Differential gene expression of senescence and SASP markers across peripheral tissues can be partially recovered by senotherapeutics. Liver, visceral adipose tissue from the peritoneum, and skeletal muscle from the gastrocnemius were isolated from mice at the end of treatment with n=3 for Control, n=4 for high-fat diet (HFD), and n=4 for HFD+JAK1/2i. All samples were assayed by qPCR for senescence genes p16Ink4a, p21Cip1, and Trp53 and SASP genes Gdf15, Il-1a, Il-6, Serpine1, and Tnfa. All bars represent mean fold change ± SEM (**A**) qPCR of isolated liver from HFD, and HFD+JAK1/2i mice with fold-change to HFD; *$p<0.05$ and **$p<0.01$ by unpaired t-test relative to HFD. (**B**) qPCR of isolated visceral adipose tissue from HFD, and HFD+ JAK1/2i mice with fold-change to HFD; **$p<0.01$ and ***$p<0.001$ by unpaired t-test relative to HFD. (**C**) qPCR of skeletal muscle from HFD, and HFD+ JAK1/2i mice with fold-change to HFD; **$p<0.01$ by unpaired t-test relative to HFD.

SUPPLEMENTARY TABLES

Supplementary Table 1. Percentages of senescent cell subpopulations in different islet cell types in control, insulin resistance (S961) and recovered insulin resistance (S961R) groups.

**Group**	**Cdkn1a$^-$/Cdkn2a$^-$**	**Cdkn1a$^+$/Cdkn2a$^-$**	**Cdkn1a$^-$/Cdkn2a$^+$**
	**Alpha cells**		
Control	86.7	13.3	0.0
S961	73.7	25.7	0.3
S961+R	82.9	16.4	0.7
	**Delta cells**		
Control	78.2	21.8	0.0
S961	62.7	37.3	0.0
S961+R	75.6	24.4	0.0
	**PP cells**		
Control	75.0	25.0	0.0
S961	67.7	31.3	1.0
S961+R	72.5	27.5	0.0

Supplementary Table 2. Selected SASP recombinant proteins for in vitro treatment of pancreatic islets

**Protein**	**Human/Mouse**	**Initial Dry Mass**	**Source**	**Catalog number**
GDF-15	Mouse	25 ug	R&D Systems	8944-GD-025
IDE	Mouse	20 ug @0.07ug/ul	OriGene	TP525567
LSAMP	Mouse	20 ug @0.15ug/ul	OriGene	TP523437
DUSP3	Mouse	20 ug @0.05ug/ul	OriGene	TP501720

Supplementary Table 3. Human Islets Donor Information

Age	Gender	T2D	BMI	Use
27	M	No	25.3	GSIS, qPCR senolytic/senomorphic, proteomics, staining
26	M	No	24.2	GSIS, qPCR senolytic/senomorphic, proteomics, staining
21	M	No	27.17	GSIS, qPCR senolytic/senomorphic, proteomics, staining
22	M	No	31.9	GSIS, qPCR senolytic/senomorphic, proteomics, staining
24	M	No	33.6	GSIS, qPCR senolytic/senomorphic, proteomics, staining
42	M	No	30.3	qPCR senolytic/senomorphic
29	M	No	26.2	qPCR senolytic/senomorphic, GSIS
64	M	No	29.1	qPCR senolytic/senomorphic, GSIS
34		No	24.6	qPCR senolytic/senomorphic
19	F	No	22.3	qPCR senolytic/senomorphic, GSIS
58	M	Yes	38.3	Perifusion
49	F	Yes	42.2	qPCR senolytic/senomorphic, GSIS
55	F	Yes	42.2	qPCR senolytic/senomorphic, GSIS, Perifusion
60	F	Yes	29.9	qPCR senolytic/senomorphic
21	M	No	28	Xenium, CODEX
71	F	No	26	Xenium, CODEX
32	M	No	28	Xenium, CODEX
54	M	No	23.9	Xenium, CODEX
28	F	No	17.7	Xenium, CODEX
22	M	No	22.4	Xenium, CODEX
23	F	No	17.2	Xenium, CODEX
40	F	No	22.97	Xenium, CODEX
37	F	No	23.5	Xenium, CODEX
47	M	No	21.8	Xenium, CODEX
69	F	No	18	Xenium, CODEX
35	M	No	23.4	Xenium, CODEX
39	M	No	24.7	Xenium, CODEX
43	M	No	14.4	Xenium, CODEX
62	F	No	23	scRNA-seq
46	M	No	23.1	scRNA-seq
42	F	No	29.3	scRNA-seq
50	F	No	28.3	scRNA-seq
57	M	No	30.9	scRNA-seq
59	M	No	24.5	scRNA-seq
58	F	No	31	scRNA-seq
64	M	No	25.5	scRNA-seq
47	M	No	28.3	scRNA-seq
57	F	No	30.2	scRNA-seq
58	M	No	28.9	scRNA-seq
34	M	No	24.6	scRNA-seq
61	M	No	29.5	scRNA-seq

Supplementary Table 4. Primer Sequences

Gene		Forward	Reverse
β-actin	Mouse	ACCGTGAAAAGATGACCCAG	GTACGACCAGAGGCATACAG
p21	Mouse	GCAGATCCACAGCGATATCC	CAACTGCTCACTGTCCACGG
p16	Mouse	CCCAACGCCCCGAACT	GCAGAAGAGCTGCTACGTGAA
p53	Mouse	CCCTGTCATCTTTTGTCCCTTC	TGGCAGAATAGCTTATTGAGG G
Lsamp	Mouse	CCCAAGACCTCCCAAGTTTAC	CCAGGTGATAACAGGTTCAGG
Igf1r	Mouse	ATTCTGATGTCTGGTCCTTCG	AGCATATCAGGGCAGTTGTC
Il1a	Mouse	TCAGCACCTTACACCTACC	GAGATAGTGTTTGTCCACATCC
Il6	Mouse	CAGAGGATACCACTCCCAAC	CAATCAGAATTGCCATTGCAC
Insulin	Mouse	CCTGTTGGTGCACTTCCTA	TCTGAAGGTCCCCGGGGCT
Mafa	Mouse	CTTCAGCAAGGAGGAGGTCAT C	GCGTAGCCGCGGTTCTT
Nkx6.1	Mouse	GAAGAGAAAACACACCAGACC	CCGTCATCCCCAGAGAATAG
Gck	Mouse	TGAAACACAAGAAACTACCCC	CCACATCCATCTCAAAGTCC
Bcl2	Mouse	GTGGTGGAGGAACTCTTCAG	GTTCCACAAAGGCATCCCAG
Casp3	Mouse	GGAGTCTGACTGGAAAGCCGA A	CTTCTGGCAAGCCATCTCCTCA
Mdm2	Mouse	CCGAGTTTCTCTGTGAAGGAGC	GTCTGCTCTCACTCAGCGATGT
Mki67	Mouse	ACCATCATTGACCGCTCCTTT	AGGCCCTTGGCATACACAAA
Pcna	Mouse	AGGGTTGGTAGTTGTCGCTG	ATTCACCCGACGGCATCTTT
TBP	Human	TGTGCACAGGAGCCAAGAGT	ATTTTCTTGCTGCCAGTCTGG
P21	Human	TGTCACTGTCTTGTACCCTTG	GCGTTTGGAGTGGTAGAAATC
P16	Human	CTTCGGCTGACTGGCTG	GCCTCCGACCGTAACTATTC
IL6	Human	AAAAGTCCTGATCCAGTTCCTG	TGAGTTGTCATGTCCTGCAG
CCL4	Human	TCCTCGCAACTTTGTGGTAG	TCCAGGTCATACACGTACTCC

Supplementary Table 5. Antibodies for Immunostaining

Antibody	Source	Catalog Number	Concentration
Anti-Insulin	Abcam	Ab195956	1:200
Anti-HMGB1	BioVision	A1005-50	1:400
Anti-Ki67	Abcam	Ab15580	1:500
Anti-PDX1	DSHB Antibodies	F6A11	1:50
Anti-Nkx6.1	DSHB Antibodies	F55A12	1:200
Anti-MafA	Abcam	Ab26405	1:200
Anti-tdTomato	Antibodies.com	A121690	1:250
488 Donkey anti-Guinea Pig IgG	Jackson ImmunoResearch Laboratories	706-545-148	1:200
594 Donkey anti-Rabbit IgG	Jackson ImmunoResearch Laboratories	711-585-152	1:200
594 Donkey anti-Goat IgG	Jackson ImmunoResearch Laboratories	705-005-003	1:200
488 Goat anti-Mouse IgG	Jackson ImmunoResearch Laboratories	115-545-003	1:200
488 Goat anti-Rabbit IgG	Jackson ImmunoResearch Laboratories	111-545-003	1:200
Anti-p21	LSBio	LS-B9242	1:50

Supplementary Table 6. Antibodies for FACS

Antibody	Source	Catalog Number	Concentration
Anti-p21	LSBio	LS-B9242	1:200
Anti-p16	Abcam	Ab54210	1:200
488 Goat anti-Mouse IgG	Jackson ImmunoResearch Laboratories	111-545-003	1:200
594 Donkey anti-Rabbit IgG	Jackson ImmunoResearch Laboratories	711-585-152	1:200

Supplementary Table 7. Raw data for Secretion index from GSIS

Fig. 2F	Low	High
P21-td TOM-	0.031	0.031
	0.036	0.033
	0.005	0.036
	0.026	0.029
	0.039	0.03
	0.037	0.022
	0.068	0.051
	0.037	0.031
	0.031	0.031
	0.035	0.035
	0.011	0.033
	0.033	0.036
	0.039	0.034
	0.009	0.063
	0.037	0.035
	0.03	0.036
	0.049	0.042
	0.063	0.046
	0.043	0.05
	0.039	0.044
	0.069	0.045
	0.013	0.062
P21-td TOM+	0.046	0.028
	0.034	0.025
	0.042	0.037
	0.04	0.05
	0.032	0.047
	0.077	0.061
	0.047	0.027
	0.1	0.062
**Fig. 4M**	**Low**	**High**
Untreated	1.701	2.604
	2.121	3.180
	3.212	3.916
Islets + Cdkn2a+ SASP factors	17.159	13.767
	11.256	9.540
	17.775	4.297
	13.094	4.015
**Fig. 5F**	**Scr-siRNA**	**p21-siRNA**
Without SASP	0.938	
	0.916	2.129
	1.231	1.508
	1.889	1.787
SASP	0.407	0.961
	1.060	0.503
	0.951	0.484
	1.187	0.505
**Fig. 6E**	**Low**	**High**
Untreated	1.701	2.604
	2.121	3.180
	3.212	3.916
Cdkn1a+ SASP factors	17.159	13.767
	11.256	9.540
	17.775	4.297
	13.094	4.015
Cdkn1a⁺ SASP + Momelotinib	18.550	20.699
	6.779	8.511
	29.759	35.614

Fig. 5C	Scr-siRNA		*p21Cip1*- siRNA	
	Low	High	Low	High
D4	0.535	0.533	0.533	0.867
	0.484	0.216	0.216	1.164
	4.075	1.486	1.486	2.412
	1.972	1.255	1.255	0.309
	5.860	2.936	2.936	0.854
	10.388	32.861		
	0.802	0.313	0.313	1.438
D6	0.272	0.973	0.973	1.283
	2.364	4.747	4.747	4.944
	2.625	0.281	0.281	0.360
	0.755	0.349	0.349	0.015
	0.555	0.570	0.570	7.890
	0.521	0.433	0.433	0.235
	1.192	0.375	0.375	0.361
D8	1.348	2.372	2.372	2.034
	5.316	0.481	0.481	0.745
	1.268	0.155		
	11.483	6.925	6.925	0.482
	8.552	4.356	4.356	6.431
	4.953	1.995	1.995	13.704
	0.410	0.291	0.291	0.444
D10	3.486	3.228	3.228	5.121
	0.751	0.283	0.283	0.494
	0.228	0.340	0.340	1.200
	3.295	3.305	3.305	0.339
	0.633	0.283	0.283	0.432
	1.605	1.110	1.110	0.063
	0.504	0.652	0.652	0.486
D12	3.069	0.378	0.378	0.531
	1.371	5.943	5.943	2.418
	1.851	2.524	2.524	0.203
	1.073	0.597	0.597	0.017
	0.376	0.226	0.226	0.318

Fig. 7G			
Control		JAK1/2i	
Low	High	Low	High
1.000	3.984	1.162	5.590
1.000	2.860	0.412	2.703
1.208	1.092	1.066	1.493
0.792	1.369	0.424	1.304
0.947	2.405	0.753	2.635
1.053	3.553	1.364	1.840
1.091	3.481	0.680	3.147
0.909	0.736	0.890	2.739
1.118	0.345	0.257	0.113
0.882	0.445	0.991	0.218
1.000	0.572	0.846	1.199
		1.563	1.658
1.639	2.986	1.904	4.158
0.400	0.949		
0.960	3.809		
1.086	0.922	4.661	3.262
0.914	0.801	1.318	1.195

Fig. 7J	Low	High
Control	4.798	3.393
	4.061	3.269
JAK1/2i (B)	5.824	3.446
	6.283	9.364
JAK1/2i (M)	7.500	7.955
	9.448	8.303

Insulin (μg/L) divided by DNA or RNA (ng/μL)

SUPPLEMENTARY METHODS

scRNASeq Analysis

Gene UMI counts in droplets were generated by aligning reads to the mouse genome (mm10). To distinguish between droplets containing cells RNA and ambient RNA, we used Monte Carlo simulations to compute *p*-values for the multinomial sampling transcripts from the ambient pool (Lun et al. 2019). First, we assumed that some barcodes correspond to empty droplets if their total UMI counts were at or below 250, 150, and 200 for CTRL, S961 and S961RECOVERED respectively. We then called cells at a false discovery rate (FDR) of 0.1%, meaning that no more than 0.1% of our called barcodes should be empty droplets on average. The number of Monte Carlo iterations determined the lower bound for the *p*-values (Phipson and Smyth 2010). There were no non-significant barcodes bounded by iterations, which indicated we didn't need to increase the number of iterations to obtain even lower *p*-values. To estimate maximum ambient contamination, we computed the maximum contribution of the ambient solution RNA to the expression profile for cell-containing droplets (Lun et al. 2019). Firstly, we estimated the composition of the ambient pool of RNA based on the barcodes with total UMI counts less than or equal to 250, 150, and 200 for each gene in CTRL, S961 and S961RECOVERED respectively. Secondly, we computed the mean ambient contribution for each gene by scaling the ambient pool by some factor. Thirdly, we computed a *p*-value for each gene based on the probability of observing an ambient count equal to or below that in cell-containing droplets based on Poisson distribution. Fourthly, we combined *p*-values across all genes using Simes' method (Simes 1986). We performed this for a range of scaling factors and identified the largest factor that yields a combined *p*-value above threshold 0.1 so that the ambient proportions were the maximum estimations.

We transformed and normalized the data for each sample using the R package sctransform (Hafemeister and Satija 2019). We performed PCA on the transformed data. Only the top 3000 variable genes were used as input.

Cell clustering. We constructed a K Nearest Neighbor (KNN) network of the cells based on the Euclidean distance in the space defined by the top principal components (PCs) that were selected by Horn's parallel analysis (Horn 1965). We refined the weights of the connection between pairs of cells based on their shared overlap in their local neighborhoods i.e. Jaccard similarity (Jaccard 1912). To cluster the cells, we applied modularity optimization techniques, i.e. the Louvain algorithm (Blondel et al. 2008).

Quality control. We visualized QC metrics as violin plots (Ilicic et al. 2016), which included the number of UMI (nCount), number of genes (nFeature), and percentage of mitochondrial UMI (percent_mt). QC metrics are reported in Main Methods section.

Preparation of islets from p21-tdTom mice for FACS of $p21^+$/$p16^+$ cell populations

p21-tdTom mice were used for islet collection and isolation was done following protocol described in Gotoh, M., *et al*. Islets were dispersed by incubation with TrypLE™ Express for 15 minutes in a 37°C water bath with vortexing every 3 minutes and resuspended in 4mL of 2% Fetal Bovine Serum (FBS) in 1x PBS. The dispersed islet cells were blocked using 1mL of Normal Goat and Normal Rat Serum from Jackson ImmunoResearch Labs in a 1:10 ratio in 1X PBS for 1 hour at 4°C. During this time the primary antibody for p16 (Anti-CDKN2A/p16INK4a antibody ab54210 from abcam) was mixed from stock into a 1:20 ratio in 1X PBS then mixed again into a 1:20 ratio in 2% FBS in PBS. Secondary antibodies were mixed into a 1:100 ratio in 2% FBS in 1X PBS. Since the $p21^+$ cells of the dTomato mice fluoresce at a red 581λ wavelength, the secondary antibody for $p16^+$ cell used was fluorescent for green at 488λ wavelength (AlexaFluor 488 Donkey anti-Rabbit). After blocking the solution was spun down at 1600RPM for 2 minutes and had the supernatant removed and replaced with 100uL of the

primary antibody solution and left to incubate for 30 minutes at 4°C. Then, the solution was spun down at 1600RPM for 2 minutes and washed with 10mL of 2% FBS in 1X PBS. The solution was spun down again at 1600RPM for 2 minutes and the supernatant was removed and replaced with 300uL of secondary antibody solution and left to incubate for 15 minutes at 4°C. The solution was spun down at 1600RPM for 2 minutes and the dispersed islet cells were resuspended 2% FBS in 1X PBS for FACS sorting using DAPI as a viability stain. Unstained samples followed all washes and solution replacements using 2% FBS in 1X PBS only.

Proteomics

After sorting, βgal-positive and βgal-negative primary mouse β-cells were plated in 96-well plates and incubated in serum-free islet media to generate conditioned media which was analyzed using SomaScan at the Genomics Proteomics Core (BIDMC). Data analysis, including, was conducted using R. Graphs were generated using GraphPad Prism.

Immunohistochemistry of mouse pancreas tissues

Paraffin-embedded blocks were prepared from tdTom+ and C57BL/6 mice pancreases and the slides were cut at 4 microns. Deparafinization step was done in the following order: Xylene-14 min; 100 EtOH- 8 min; 95% EtOH-6 min; 75% EtOH-10 min and ddH_2O 5 min. Slides were washed for 10 min in 2% Lamb serum containing PBS. Later avidin was applied for 30 min followed by PBS (2% Lamb) and biotin for 30 min. After another washing step with PBS (2% Lamb) slides were incubated in Normal Donkey Serum (2% in PBS) for 30 minutes and then specific primary antibodies were incubated overnight at 4°C. On day 2 the slides were brought to RT for 1 hour then washed with PBS (2% Lamb) for 10 min. Afterwards respective secondary antibodies were applied for 1 hour at RT. Washing step with PBS followed then the second primary antibody was applied and incubated overnight at 4°C. On day 3 the slides were brought to RT for 1 hour then washed with PBS (2% Lamb) for 10 min. Afterwards respective secondary antibodies were applied for 1 hour at RT. Washing step with PBS followed then the sections were mounted with Fluoroshield with DAPI mounting media. Images were taken using confocal mode on a Zeiss LSM 710 microscope.

Whole Pancreas Preparation

All the whole pancreases were procured by the University of Texas Health San Antonio from IRB-exempt brain-dead donors and then shipped to Joslin Diabetes Center, Harvard Medical School. The pancreata were obtained at University Hospital Center for Life, which is managed in partnership between University Health Transplant Institute (UHTI) and Texas Organ Sharing Alliance (TOSA), who is the local Organ Procurement Organization. TOSA has jurisdiction over the donors, and an approval was received from their Advisory Board to receive organs intended for research. Inclusion criteria: Age range 20-60+. Exclusion criteria: type 2 diabetes, pancreatic malignancy, pancreatitis, use of agents with senolytic activity. The pancreases were cut into the head, body, and tail of the locations along the pancreas carcinoma guideline, cut into 4mm thickness, and placed into order paraffin or frozen cassettes. The paraffin cassettes were fixed in 4% paraformaldehyde for 24 hrs and processed for paraffin embedding at Joslin Histology Core. The frozen cassettes were molded with O.C.T. compound (Fisher Scientific No.23-730-571) and frozen in the cold 2-methylbutane in a beaker surrounded by dry ice and 100% ethanol (Fisher Scientific No.60-048-072) bath followed by drying and storage at -80°C. At least three paraffin cassettes per each location of head, body, and tail were shipped to The Jackson Laboratory; all the procedures were done at 4°C and avoided exposure to direct light. A detailed protocol can be found in https://www.protocols.io/view/kapp-sen-tmc-whole-pancreas-preparation-4r3l224nql1y/v1?step=1

Doxorubicin Treatment In vitro

Isolated islets were exposed to 200 nM doxorubicin for 24 h. After treatment, the drug-containing medium was removed, islets were washed thoroughly and then incubated in fresh culture medium for an additional 48 h wash out period prior to downstream assays.

scRNASeq of Human Islets

Pancreatic islets from Prodo Lab were digested with StemPro™ Accutase™ Cell Dissociation Reagent at a concentration of 1 ml/1,000 islets for 10 minutes at 37°C. All dissociated cells were filtered through a 40 µm Flowmi® Cell Strainers (SIGMA) and counted using AO/PI (acridine orange/propidium iodide) Cell Viability Kit for Luna-FL automated cell counter. Cells were fixated prior to scRNAseq according to 10X Genomics protocol CG000478. After determining cell concentration of the fixed sample by AO/PI Cell Viability Kit for Luna-FL automated cell counter pre-warmed Enhancer (10x Genomics PN-2000482) was added and samples stored for up to 1 week. For single cell library preparation and sequencing the cell viability was assessed on a LUNA FX7 automated cell counter (Logos Biosystems), and up to 12,000 cells from each suspension were loaded onto one lane of a 10x Genomic Chip [G]. Single cell capture, barcoding, and library preparation were performed using the 10x Genomics Chromium X platform [CITE https://www.nature.com/articles/ncomms14049] version 3.1 NEXTGEM chemistry and according to the manufacturer's protocol (#CG000315). cDNA and libraries were checked for quality by Tapestation 4200 (Agilent) and Qubit Fluorometer (ThermoFisher), quantified by KAPA qPCR, and sequenced on an Illumina NovaSeq X+ 10B 100 cycle flow cell lane, with a 28-10-10-90 asymmetric read configuration, targeting 6,000 barcoded cells with an average sequencing depth of 50,000 reads per cell. Illumina base call files for all libraries were converted to FASTQs using bcl2fastq v2.20.0.422 (Illumina) and FASTQ files associated with the gene expression libraries were aligned to the GRCm38 reference assembly with vM23 annotations from GENCODE (10x Genomics mm10 reference 2020-A) using the version 8.0.0 Cell Ranger multi pipeline (10x Genomics).

FFPE Block DV200 Evaluation

Prior to the spatial transcriptomics assay, the FFPE blocks were assessed for RNA quality by DV200 scores. Two to four 5µm FFPE sections were collected in RNAse free conditions and RNA was extracted using Qiagen's RNeasy Micro Kit (Cat. No. / ID: 74004). RNA quality was then quantified using a Tapestation High Sensitivity RNA ScreenTape (Agilent). DV200 scores were calculated as the percentage of the RNA sample above 200 bases in length, as determined using Agilent TapeStation Analysis software. DV200 score of more than 50% was achieved for all FFPE blocks

CODEX/ PhenoCycler Slide Preparation, Setup, and Data Acquisition

The slides were baked at 60°C for 30 minutes. After baking, slides were immersed in xylene twice for 5 minutes each, followed by incremental rehydration in 100%, 95%, 70%, and 50% ethanol for 3 minutes each. The slides were then immersed in water for 3 minutes and moved to 1x antigen retrieval buffer (pH 9.0) (AR9, Akoya Biosciences), for antigen retrieval at 100 °C for 15 minutes using the TintoRetriever (BioSB). Following antigen retrieval, the slides were cooled in the retrieval buffer to RT and washed in nuclease-free water for 5 minutes. Slides were then processed according to the PhenoCycler-Fusion User Guide (PD-000011 REV M, Akoya Biosciences), starting from step 4 on page 49. Briefly, the slides were washed in Hydration Buffer, equilibrated in Staining Buffer, and incubated overnight at 4°C with a 38-marker antibody cocktail prepared in Blocking Buffer. The slides were subsequently washed in Staining Buffer, gently fixed with Post-Staining Fixing Solution, washed in 1x PBS, and incubated in ice-cold methanol for 5 minutes. The slides were then washed in 1x PBS, fixed with Final Fixative

Solution for 20 minutes, washed three times in 1x PBS, and immersed in Storage Buffer prior to the PhenoCycler Fusion run.
The experimental protocol was set up using the PhenoCycler Experiment Designer (Version 2.1.0, Akoya Biosciences). A reporter plate containing fluorescently labelled barcode reporters, as per the experimental design, was prepared following instructions on page 73 of the PhenoCycler-Fusion User Guide (PD-000011 REV M, Akoya Biosciences). Slides were prepared for the PhenoCycler-Fusion instrument according to the steps outlined in the PhenoImager-Fusion User Guide (PD-000001 Rev N, Akoya Biosciences). Briefly, the slide was moved from Storage Buffer to 1x PBS and incubated for 10 minutes. After incubation, a Flow Cell (Akoya Biosciences) was attached to the sample slide using the Flow Cell Assembly Device (Akoya Biosciences). The slide with the attached Flow Cell (Sample Flow Cell) was then placed in 1x PhenoCycler buffer for 10 minutes. PhenoCycler Fusion software (Version 2.2.0) was used to set up the imaging run on the PhenoCycler-Fusion, following the steps on page 57 of the PhenoImager-Fusion User Guide (PD-000001 Rev N, Akoya Biosciences). Reagents were prepared and loaded into the appropriate reagent reservoirs on the instrument, and the pre-prepared reporter plate was loaded into the PhenoCycler. A new PhenoCycler run was initiated using the experimental protocol design. A blank flow cell was loaded into the Flow Cell Slide Carrier, and all software prompts during the pre-flight routine were followed. The Sample Flow Cell was then loaded into the carrier and a leak check was performed. Scan regions were selected following automated sample finding and imaging was started. Upon completion, the Sample Flow Cell was placed in Storage Buffer at 4°C. The generated QPTIFF data file was used for downstream image analysis.

Xenium
Genes for Xenium panels were selected based on literature review and initial scRNAseq and Visium analysis. Given a list of gene targets ordered by level of importance, a custom 300 gene panel was designed using the 10x Genomics Xenium Panel Designer web-based tool (cloud.10xgenomics.com,) using Visium CytAssist and Fixed scRNAseq (10x Genomics) data generated from matched samples as a reference to gauge anticipated transcript abundance and ubiquity. Due to the highly abundant genes targeted, such as INS, GCG, and SPINK2, the panel design process was run in an iterative fashion where (a) each target initially excluded due to high expression was reassigned 1 probe, then (b) the designer was run to generate probeset assignments for all other genes to accommodate the high-expressers. This process was repeated until the panel designer no longer excluded desired highly expressed genes.
FFPE Tissue sections of 5μm thickness were placed on the Xenium slide (10x Genomics, Cat. No. 1000660) such that 2 sections filled the 10.45 x 22.45mm capture area. The tissue was deparaffinized, permeabilized, de-crosslinked, and stained with probes according to manufacturer specifications (protocol CG000580, user guide CG000582). Slides were loaded onto the Xenium Analyzer and in situ gene expression data were generated according to the manufacturer's user guide (CG000584, RevG). After the Xenium run, data were exported from the analyzer for analysis, and Xenium slides were subject to H&E staining according to the manufacturer demonstrated protocol (CG000613) and imaging via Nanozoomer SQ (Hamatsu). Following Xenium, multiplexed antibody-based immunofluorescence staining and imaging was performed using the Phenocycler-Fusion (Akoya Biosciences) according to a custom protocol (https://dx.doi.org/10.17504/protocols.io.q26g71rwqgwz/v1)].

REFERENCES

Blondel, Vincent D, Jean-Loup Guillaume, Renaud Lambiotte, and Etienne Lefebvre. 2008. “Fast Unfolding of Communities in Large Networks.” *Journal of Statistical Mechanics: Theory and Experiment* 2008 (10): P10008. https://doi.org/10.1088/1742-5468/2008/10/P10008.

Hafemeister, Christoph, and Rahul Satija. 2019. “Normalization and Variance Stabilization of Single-Cell RNA-Seq Data Using Regularized Negative Binomial Regression.” *Genome Biology* 20 (1): 296. https://doi.org/10.1186/s13059-019-1874-1.

Horn, J L. 1965. “A RATIONALE AND TEST FOR THE NUMBER OF FACTORS IN FACTOR ANALYSIS.” *Psychometrika* 30 (June): 179–85. https://doi.org/10.1007/{BF02289447}.

Ilicic, Tomislav, Jong Kyoung Kim, Aleksandra A Kolodziejczyk, Frederik Otzen Bagger, Davis James McCarthy, John C Marioni, and Sarah A Teichmann. 2016. “Classification of Low Quality Cells from Single-Cell RNA-Seq Data.” *Genome Biology* 17 (February): 29. https://doi.org/10.1186/s13059-016-0888-1.

Jaccard, Paul. 1912. “The Distribution of the Flora in the Alpine Zone.” *The New Phytologist* 11 (2): 37–50. https://doi.org/10.1111/j.1469-8137.1912.tb05611.x.

Lun, Aaron T L, Samantha Riesenfeld, Tallulah Andrews, The Phuong Dao, Tomas Gomes, participants in the 1st Human Cell Atlas Jamboree, and John C Marioni. 2019. “EmptyDrops: Distinguishing Cells from Empty Droplets in Droplet-Based Single-Cell RNA Sequencing Data.” *Genome Biology* 20 (1): 63. https://doi.org/10.1186/s13059-019-1662-y.

Phipson, Belinda, and Gordon K Smyth. 2010. “Permutation p-Values Should Never Be Zero: Calculating Exact p-Values When Permutations Are Randomly Drawn.” *Statistical Applications in Genetics and Molecular Biology* 9 (October): Article39. https://doi.org/10.2202/1544-6115.1585.

Simes, R. J. 1986. “An Improved Bonferroni Procedure for Multiple Tests of Significance.” *Biometrika* 73 (3): 751–54. https://doi.org/10.1093/biomet/73.3.751.
